# The chemokine monocyte chemoattractant protein-1/CCL2 is a promoter of breast cancer metastasis

**DOI:** 10.1038/s41423-023-01013-0

**Published:** 2023-05-19

**Authors:** Teizo Yoshimura, Chunning Li, Yuze Wang, Akihiro Matsukawa

**Affiliations:** Department of Pathology and Experimental Medicine, Graduate School of Medicine, Dentistry and Pharmaceutical Sciences, Okayama University, 2-5-1 Shikata, Kita-ku, Okayama, 700-8558 Japan

**Keywords:** Breast cancer, chemokines, chemokine receptors, metastasis, macrophages, Breast cancer, Chemokines

## Abstract

Breast cancer is the most prevalent cancer worldwide, and metastasis is the leading cause of death in cancer patients. Human monocyte chemoattractant protein-1 (MCP-1/CCL2) was isolated from the culture supernatants of not only mitogen-activated peripheral blood mononuclear leukocytes but also malignant glioma cells based on its in vitro chemotactic activity toward human monocytes. MCP-1 was subsequently found to be identical to a previously described tumor cell-derived chemotactic factor thought to be responsible for the accumulation of tumor-associated macrophages (TAMs), and it became a candidate target of clinical intervention; however, the role of TAMs in cancer development was still controversial at the time of the discovery of MCP-1. The in vivo role of MCP-1 in cancer progression was first evaluated by examining human cancer tissues, including breast cancers. Positive correlations between the level of MCP-1 production in tumors and the degree of TAM infiltration and cancer progression were established. The contribution of MCP-1 to the growth of primary tumors and metastasis to the lung, bone, and brain was examined in mouse breast cancer models. The results of these studies strongly suggested that MCP-1 is a promoter of breast cancer metastasis to the lung and brain but not bone. Potential mechanisms of MCP-1 production in the breast cancer microenvironment have also been reported. In the present manuscript, we review studies in which the role of MCP-1 in breast cancer development and progression and the mechanisms of its production were examined and attempt to draw a consensus and discuss the potential use of MCP-1 as a biomarker for diagnosis.

## Introduction

It has been thirty-five years since the isolation and purification of the first chemokine, monocyte-derived neutrophil chemotactic factor (known as interleukin-8 or CXCL8), was first described [1]. Since then, many chemokines have been identified by biology-based protein purification, cDNA cloning, or data mining, and they have been found to form a large family of leukocyte chemotactic proteins comprising four subfamilies. Receptors for each chemokine have also been cloned [2, 3]. Although the discovery phase of chemokine research is mostly completed, chemokines and their receptors continue to attract scientific interest because they could be targets for clinical interventions in human diseases, including cancer. In tumor microenvironments (TMEs), various chemokines are coordinately produced and regulate the infiltration of leukocyte subsets with anti- or protumor activity in a spaciotemporal manner, and the contributions of chemokines to cancer development and progression have been described in many excellent reviews [4–8].

Monocyte chemoattractant protein-1 (MCP-1/CCL2) was purified in 1989 and was the second chemokine isolated based on its in vitro monocyte chemotactic activity [9]. MCP-1 was purified from the culture supernatants of not only activated peripheral blood mononuclear leukocytes [10] but also malignant glioma cells [11] and was subsequently found to be identical to the factor previously described as tumor cell-derived chemotactic factor (TDCF) [12], which is hypothesized to be responsible for the infiltration of blood monocyte-derived macrophages, the so-called tumor-associated macrophages (TAMs), into tumors. Many human cancer cell types have been found to constitutively produce MCP-1 in culture, and the expression of MCP-1 has been detected in many human cancer tissues, including gliomas [13, 14], meningioma [15], ovarian cancer [16], and lung cancer [17]. When MCP-1 was identified, the role of TAMs, namely, as cytotoxic antitumor cells or cancer-promoting cells (protumor), was still controversial [18]. Early studies aiming to use MCP-1 as an inducer of macrophage-mediated tumor cell killing suggested that MCP-1 is useful for inducing antitumor activities [19–23]. However, the results of later work strongly suggested that MCP-1 acts as a tumor-promoting factor [24].

Breast cancer (BC) is the most prevalent cancer worldwide, and metastasis is the leading cause of death [25, 26]. BC is a highly heterogeneous malignant disease that can be caused by a variety of distinct genetic alterations in mammary epithelial cells, leading to vastly different disease manifestations in individual patients [27]. The contribution of several chemokines to the progression of BC has been reported, but MCP-1 is the most extensively studied chemokine in human BC tissues, human BC cell lines, and animal BC models, and the results strongly suggest the involvement of MCP-1 in the lung metastasis of BC. Here, we review studies focusing on MCP-1 and BC and then attempt to draw a consensus. We also discuss the potential use of MCP-1 as a biomarker for diagnosis and a therapeutic target.

Several terms were originally given to each chemokine, but the use of a systemic nomenclature was proposed at the Keystone Symposia in 1999 [28]. The term CCL2 is now widely used for this chemokine, and readers of this review are likely more accustomed to this systemic term. Therefore, we use the term CCL2 in the rest of this review.

## Detection of CCL2 in human BC tissues and its production by BC cells

### Detection in human BC tissues by immunohistochemistry (IHC)

To characterize CCL2 expression in invasive ductal carcinomas (IDCs), Valkovic et al. [29] first evaluated the expression of CCL2 in the parenchymal and stromal cells of 27 IDC cases (Table 1). CCL2 was detected in the parenchyma in 15 of 27 (56%) ductal carcinomas. Various levels (high to undetectable) of CCL2 were detected in the tumor epithelium. Histologically, CCL2-negative tumors tended to be well differentiated, whereas CCL2-positive tumors exhibited low levels of differentiation. CCL2 immunoreactivity was also detected in TAMs defined by the presence of CD68 expression in 23 of 27 (85%) tumors and in endothelial cells (ECs) in 11 of 27 (41%) tumors. These results indicated that both the parenchymal and stromal components of human IDC express CCL2 in vivo. The findings also suggested that CCL2 expression in the tumor parenchyma is correlated with the histological grade of ductal invasive breast carcinoma. The same authors extended their study by analyzing 97 IDC cases. CCL2 immunoreactivity was present in tumor cells in 17 of 97 (18%) tumors but also in TAMs, fibroblasts, and ECs in the majority of tumors. There was no significant correlation between CCL2 expression in the tumoral epithelium and tumor size, histological grade, mitotic activity index (MAI), lymph node status, or level of TAMs [30].

**Table 1:** Detection of CCL2 in human BC tissues by IHC and correlations between CCL2 positivity and the level of TAM infiltration and cancer progression

Authors [Ref]	BC cases	CCL2^+^ cells	Biological significance
Valković, et al. [29]	Invasive ductal carcinoma (27 cases)	Parenchyma (15/27), TAMs (CD68^+^) (23/27), ECs (11/27)	CCL2 expression in parenchyma correlated with the histological grade.
Ueno, et al. [31]	Invasive ductal carcinoma (151 cases)	Cancer cells, monocytic cells, fibroblastic cells	CCL2 concentration was correlated significantly with the level of VEGF, TP, TNFα. CCL2 was associated significantly with TAM accumulation. High expression of CCL2 was a significant indicator of early relapse.
Saji, et al. [32]	Primary BC (230 cases)	Cancer cells (174/230), tumor stroma (117/230); TAMs (CD68^+^), Lymphocytes, Fibroblasts, SMCs	Stromal CCL2 correlated with lymphatic invasion, venous invasion.
Chavey, et al. [33]	Primary BC (105 cases)	No data available	CCL2 expression was higher in ER-negative tumors and PR-negative tumors. CCL2 was abundant in high-grade tumors. CCL2 level was correlated with marked leukocyte infiltration.
Valković, et al. [30]	Invasive ductal carcinoma (124 cases, 97 cases for IHC)	Parenchyma (17/97), TAMs (CD68^+^), Fibroblasts, ECs in the majority of tumors	CCL2 expression in parenchyma did not correlate with tumor size, histological grade, mitotic activity index, and lymph node involvement. CCL2 expression did not correlated with TAM accumulation.
Fujimoto, et al. [34]	Invasive ductal carcinoma (128 cases)	TAMs (CD68^+^) were the major CCL2^+^ cells	Stromal CCL2 correlated with TAM infiltration, relapse-free survival.
Soria et al. [35]	DCIS (30 cases) and IDC (58 cases)	Mostly cancer cells	CCL2 was co-expressed with CCL5, TNFα, and IL-1β in tumor cells.
Katanov, et al. [36]	IDC (case number not available)	Cancer cells and fibroblasts were the major CCL2^+^ cells	inflammation–stroma interactions induce CCL2 expression by fibroblasts via NF-kB activation.
Li, et al. [38]	ER^+^ BC (100 cases)	TAMs (CD163^+^)	Stromal CCL2 correlated with poor prognosis and promoted endocrine resistance.
Heiskala, et al. [41]	Primary breast cancer (52 cases), core needle biopsies and resected tumors	Malignant epithelial cell staining was stronger in CNB, whereas the fraction of CCL2-positive lymphocytes was higher in resected tumors	Trauma by CNB induces inflammation in cancer stroma.

Ueno et al. evaluated the local expression of cytokines, chemokines, and angiogenic factors in 151 primary BC tissues [31]. The CCL2 concentration in tumor homogenates was positively correlated with the level of cytokines with angiogenic activity, such as vascular endothelial growth factor (VEGF), tumor necrosis factor (TNF) α, CXCL8 and thymidine phosphorylase (TP). The level of CCL2 was significantly associated with the level of TAM accumulation. According to the IHC results, CCL2 expression was observed in both CD68^+^ TAMs and tumor cells. Prognostic analysis revealed that high expression of CCL2 and VEGF was a significant indicator of early relapse and that combined VEGF and CCL2 status was an independent prognostic indicator in BC. From these findings, it was concluded that the interaction between the immune system and angiogenesis is important for the progression of human BC and that CCL2 may play an important role in the regulation of angiogenesis and the immune system.

Saji et al. examined 230 primary IDC cases and reported that 117 (51%) specimens had intensive CCL2-positive staining in tumor cells. The expression of CCL2 in tumor cells had a significant correlation with the expression of TP and membrane type 1-matrix metalloproteinase (MMP). In addition, CCL2 expression tended to be associated with the accumulation of CD68^+^ TAMs and microvessel density, as defined by positive staining for von Willebrand factor. CCL2 expression in TAMs was correlated significantly with the histological vessel invasion of tumor cells. These findings suggested that CCL2 may play key roles in macrophage recruitment, the expression of angiogenic factors, and the activation of MMPs in patients with BC [32].

Chavey et al. screened 17 members of the cytokine family, including CCL2, in 105 BC tissues and 13 healthy breast biopsies by a combination of IHC and fluorescence in situ hybridization (ISH) analysis. CCL2 levels were very low in healthy breast tissue samples but higher in BC tissues. The levels of CCL2 were significantly higher in estrogen receptor (ER)-negative tumors than in ER-positive tumors. CCL2 was more abundant in progesterone receptor (PR)-negative tumors than in PR-positive tumors and was abundant in high-grade tumors. High expression levels of CCL2, CXCL8, and CCL4 were correlated with strong infiltration of inflammatory cells, such as B cells, T cells, and CD68^+^ macrophages [33].

Fujimoto et al. examined 128 IDC cases by IHC and detected a significant positive correlation between stromal CCL2 expression and the number of TAMs. Positive CCL2 staining of stromal cells, but not tumor cells, had a significant correlation with relapse-free survival [34].

Soria et al. investigated the expression of CCL2 along with CCL5, TNFα, and interleukin (IL)-1β by IHC in breast samples of 38 healthy individuals and 88 BC patients (30 ductal carcinoma in situ [DCIS] patients, 23 IDC patients who remained free after treatment and 35 IDC patients who relapsed with metastases or local tumors or who died of BC). All cytokines/chemokines were expressed at very low levels in normal breast epithelial cells, but their expression levels were significantly elevated in tumor cells of the three groups of cancer patients. All four factors were only minimally detected in infiltrating leukocytes of all groups of patients but were clearly present in breast tumor cells, with mainly a cytoplasmic staining pattern [35]. CCL2-positive staining was also found in fibroblasts in IDC in a different study by the same research group [36].

Wang et al. examined the level of CCL2 in 205 BC cases by IHC and compared its expression levels among five BC subtypes: basal-like, HER2, luminal A, luminal B, and normal breast-like. CCL2 expression was negatively associated with the expression of both ER and PR in breast tumor tissues and more strongly associated with the expression of PR. There was no information as to the cell types expressing CCL2 [37].

Li et al. performed IHC on tissue sections from invasive ER-positive BC patients. High expression of CCL2 was correlated with the infiltration of CD163^+^ TAMs, and patients with high CCL2 expression levels presented shorter progression-free survival than those with low CCL2 expression levels [38].

Core needle biopsy (CNB) is clinically used to verify the diagnosis of malignancy before surgery in BC patients [39], but there is a risk of enhancing tumor development by this procedure. In a mouse experiment, a needle biopsy significantly increased the frequency of distant metastases, and inflammation induced by CNB was determined to be responsible [40]. Heiskala et al. compared the levels of CCL2 and markers expressed by monocytes/macrophages (CD163, CD14, and the CCL2 receptor CCR2) in samples from CNBs to those from the corresponding resected tumors from 52 patients with primary BC. CCL2, CCR2, CD163, and CD4 were all widely expressed in both CNB and resected tumor samples. CCL2 expression was detected in malignant epithelial cells and in a fraction of lymphocytes in close contact with cancer cells [41].

The findings introduced here are summarized in Table 1. In brief, CCL2 has been detected in both cancer cells and stromal cells, including TAMs, fibroblasts, and ECs (lymphocytes in one study). CCL2-positive staining is most consistently detected in TAMs, suggesting that TAMs are one of the major CCL2 sources in BC microenvironments. With regard to the association between CCL2 expression and BC subtypes, no significant correlation between the level of CCL2 and the status of ER or PR expression was found in two studies [31, 32], whereas a negative association of CCL2 expression with the expression of both ER and PR was detected in one study [37]. Further studies are necessary to determine whether there is a clear association between the level of CCL2 staining and BC subtypes.

### Production of CCL2 by human and mouse BC cells in vitro

A wide variety of BC cell lines have been established from BC tissues and used for BC-related studies [42, 43]. BC is highly heterogeneous and categorized into groups, for example, luminal A, luminal B, HER2-positive, triple-negative/basal type, and triple-negative/mesenchymal-like type; thus, established cell lines derived from different BCs can also be categorized into those groups [42, 44]. Saji et al. examined the capacity of CCL2 production in five types of human BC cells in vitro by ELISA. Three cell types, HBC-5, BSY-1, and HTB-26, produced significant levels of CCL2, whereas CCL2 production was not detected in two cell types, MCF-7 and MDA-MB-231 (Table 2) [32]. Notably, the cell line name “HTB-26” is used for MDA-MB-231 cells (https://www.atcc.org/products/htb-26), and it is unclear whether those two cell lines used in the study were identical.

**Table 2:** Production of CCL2 by human BC cell lines. ND, not detected

Authors [Ref]	Cell lines	CCL2 production
Saji, et al. [32]	HBC-5, BSY-1, HTB-26	+
	MCF-7, MDA-MB-231	ND
Nam, et al. [45]	MDA-MB-231	30 ng/10^6^ cells/48 h
	T47D	7 pg/10^6^ cells/48 h
Dwyer, et al. [47]	MDA-MB-231	555 pg/ml
	T47D	97n pg/ml
Wang, et al. [45]	MCF7	1310.56 pg/ml/24 h
	BT474	1389.13 pg/ml/24 h
	MDA-MB-468	1720.80 pg/ml/24 h
	SKBR3	1799.19 pg/ml/24 h
Dutta, et al. [46]	BT549, HCC1395	>7000 pg/ml/10^6^ cells
	HCC1937, HCC70, HCC1806, T47D, MDA-MB-231	4000-6000 pg/ml/10^6^ cells
	MCF-7, SK-BR-3	2000 pg/ml/10^6^ cells
Li, et al. [38]	MCF-7	ND

*ND* not detected

The lack of CCL2 production by MCF-7 cells was also reported by Li et al. [38]. In a study by Nam et al., a high level of CCL2 was secreted by MDA-MB-231 cells (approximately 30 ng/10^6^ cells/48 h) and a low level (approximately 7 pg/10^6^ cells/48 h) by T47D cells [45]. In a study by Dutta et al. [46], the levels of *CCL2* mRNA expression and CCL2 protein production were examined in HCC1937, HCC1395, HCC70, and HCC1806 (categorized as triple-negative [TN] BC/basal-type cells) and BT-549 and MDA-MB-231 (categorized as TNBC/mesenchymal-like cells) cells. MCF-7, BT474, and T47D cells represented luminal-type cells, and SKBR3 cells represented Her2-positive or Her2-enriched cancer cells. The highest *CCL2* mRNA levels were found in BT549 and HCC1395 (TNBC/mesenchymal-like) cells. The secreted CCL2 protein levels were higher in most TNBC cell types than in other cell types. In contrast to the data by Saji et al. and Li et al., approximately 2000 pg/ml/10^6^ cells and 3000 pg/ml/10^6^ cells of CCL2 were detected in the culture supernatants of MCF-7 and MDA-MB-231 cells, respectively. Dwyer et al. also detected 555 pg/ml and 97 pg/ml CCL2 in the culture supernatants of MDA-MB-231 and T47D cells [47]. Wang et al. reported that MCF-7, BT474, MDA-MB-468, and SKBR3 cells secreted approximately 1310.56, 1389.13, 1720.80, and 1799.19 pg/ml CCL2 in 24 h, respectively [37]. Thus, many types of cultured human BC cells produce significant levels of CCL2. The capacity of CCL2 production by certain cell lines, such as MCF-7 and MDA-MB-231, was different among the studies, which may be due to different culture conditions. There is also evidence that dramatic genetic and epigenetic changes occur during initial cell line establishment and subsequent serial passaging [43]. This could be another explanation for the detection of different CCL2 levels by the same cell line in different laboratories.

**Fig. 1:**
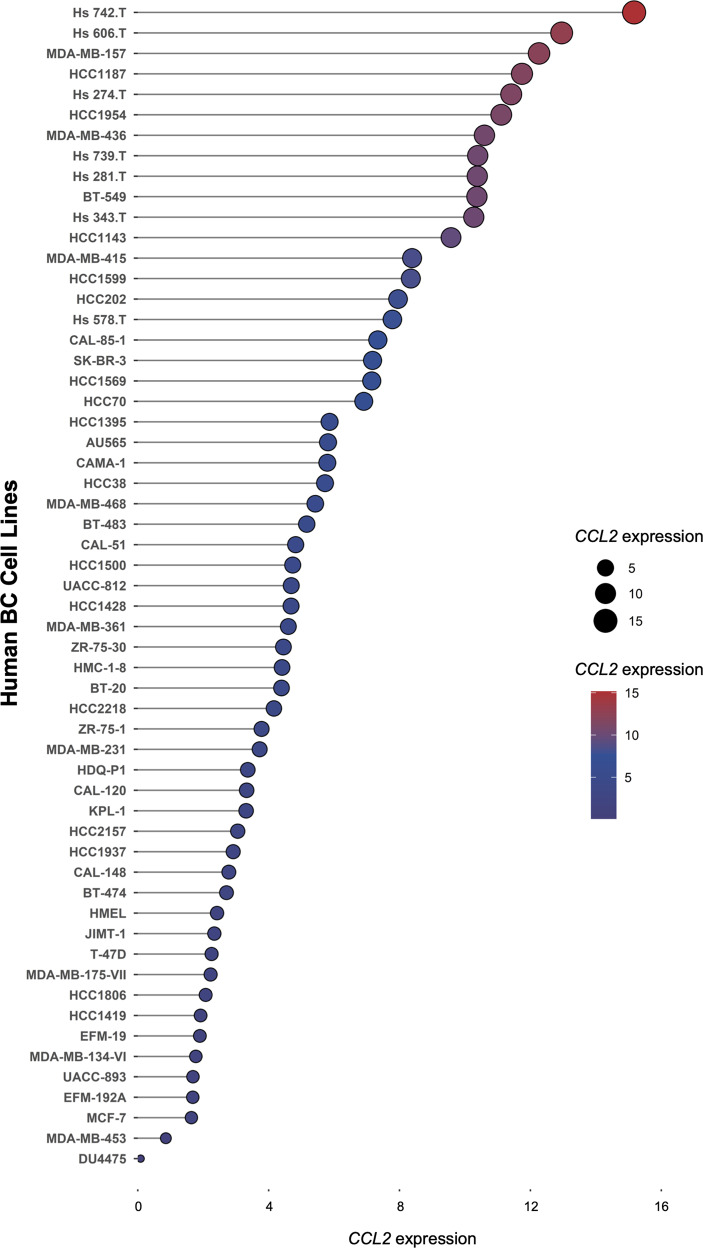
The expression distribution of *CCL2* mRNA in human BC cells. The abscissa represents the expression distribution of mRNA, and the ordinate represents different cell types. Different colors and the size of dots represent expression. The cell line mRNA expression matrix was obtained from the CCLE dataset (https://portals.broadinstitute.org/ccle) [48]. The analysis was constructed with the R v4.0.3 software package ggplot2 (v3.3.3)

We analyzed the levels of *CCL2* expression in 57 human BC cell lines using the RNA-seq data available from the CCLE dataset (https://portals.broadinstitute.org/ccle) [48] (Fig. 1). Consistent with the results of previous studies, in vitro cultured human BC cells were shown to express *CCL2* mRNA at various levels.

Mouse BC cell line cells also produce CCL2. Lymphocyte antigen 6 family member E.1 (Ly6E.1) is highly expressed in murine tumor cells with a highly malignant phenotype. Mouse DA3 BC cells expressing a high level of Ly6E.1 (Ly6^hi^) were more highly tumorigenic than the same cells expressing low levels of this membrane protein (Ly6^lo^) [49]. Neumark et al. found that Ly6^high^ DA3 cells produced a higher level of CCL2 than Ly6^low^ DA3 cells [50]. Additionally, 4T1 cells [51] and EO771 cells [52] constitutively produce CCL2.

**Fig. 2:**
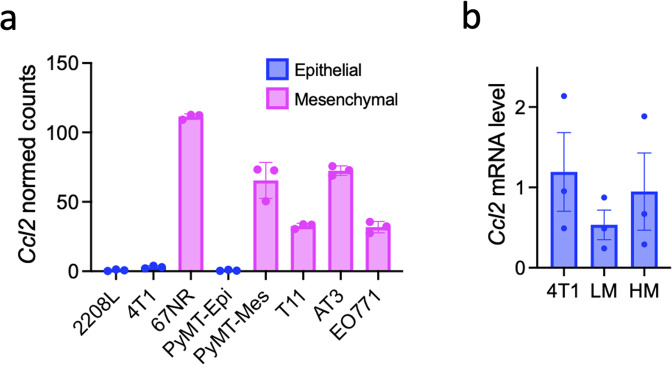
The expression of *Ccl2* mRNA in mouse BC cells. **a** The expression distribution of *Ccl2* mRNA in 8 mouse BC cell types. The raw RNA sequencing data of 8 mouse BC cell types were obtained from the GEO database (ID: GSE104765). The R package edgeR [58] was used to scale the raw data and generate scaled counts. The formula calcNormFactors (method = ‘TMM’) [59] of the R package edgeR was used to scale every single sample to eliminate the variance in sequencing depth. The scaled data were extracted using the formula cpm() in the R package edgeR, and the scaled count values of *Ccl2* were applied to build the graph using the R package ggplot2 [60]. **b** The expression of *Ccl2* mRNA in two 4T1 cell subtypes, HM.4T1 and LM.4T1, as determined by RT‒qPCR

Kim et al. [53] performed RNA sequence analysis on 8 mouse BC cell lines, namely, 2208 L (derived from a BALB/c p53-null tumor), 4T1, 67NR (BALB/c tumor), PyMT-Epi, PyMT-Mes (C57BL/6 MMTV-PyMT tumor), T11 (BALB/c p53-null tumor), AT-3 (C57BL/6 MMTV-PyMT tumor) [54] and EO771 (C57BL/6 tumor) [55, 56]. 4T1 (metastatic) and 67NR (nonmetastatic) cells are subpopulations of a single mouse mammary tumor [57]. PyMT-epithelial and PyMT-mesenchymal cells are also subpopulations of a single mouse mammary tumor. The expression profile of a panel of EMT-related genes was used to classify three cell lines, 2208L, 4T1, and PyMT-Epi, as epithelial, whereas 67NR, PyMT-Mes, T11, AT-3, and EO771 were classified as mesenchymal. We analyzed the expression of *Ccl2* mRNA in these 8 cell lines using the GEO database. The raw counts were first extracted from GSE104765. The R package edgeR [58] was next used to scale the raw data and generate scaled counts. The formula calcNormFactors (method = ‘TMM’) [59] of the R package edgeR was used to scale every single sample to eliminate the variance in sequencing depth. The scaled data were extracted using the formula cpm() in the R package edgeR, and the scaled count values of *Ccl2* were applied to build the graph using the R package ggplot2 [60]. Interestingly, all epithelial BC cells expressed low levels, whereas all mesenchymal BC cells expressed high levels of *Ccl2* mRNA (Fig. 2a). 4T1 is not a single clone and comprises a mixture of subclones [61]. We isolated two 4T1 subclones, LM-4T1 and HM-4T1, with low and high metastatic capacities, respectively [62]. Compared to LM-4T1 cells, HM-4T1 cells express a lower level of E-cadherin and a higher level of Snail, suggesting that HM-4T1 cells are more mesenchymal. We evaluated the level of *Ccl2* mRNA in 4T1, LM-4T1, and HM-4T1 cells by reverse transcription-quantitative polymerase chain reaction (RT‒qPCR) and found that the *Ccl2* mRNA level was slightly higher in HM-4T1 cells than in LM-4T1 cells (Fig. 2b). Although the differences were not statistically significant, it appears that the level of *Ccl2* expression varies even among 4T1 subclones.

**Fig. 3:**
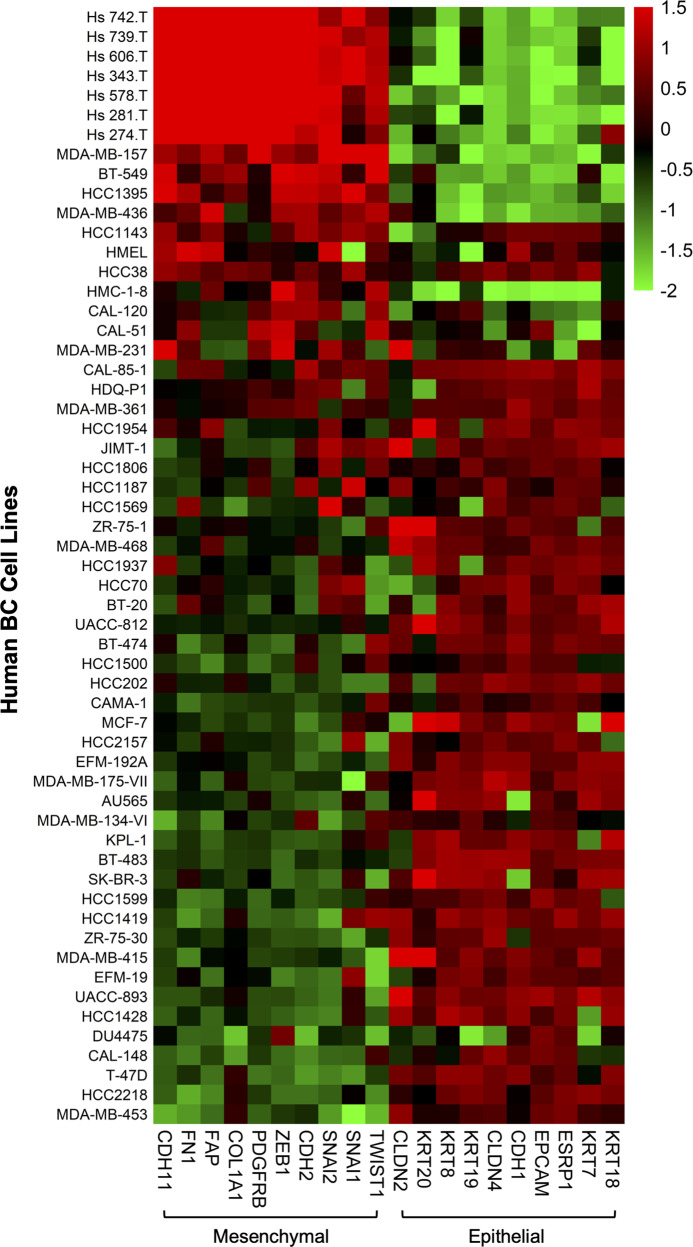
Heatmap showing the expression level of a panel of EMT-related genes across 59 human BC cell types. The count value of each gene was obtained as in Fig. 2. A heatmap was generated using the pheatmap package (version 1.0.12) [200]. The color scale indicates the z scores of regularized log-transformed data across columns

To examine whether the same correlation can be found among human BC cells, we evaluated the EMT status of 57 types of human BC cells (Fig. 1) by analyzing RNA sequencing data. Cells in high EMT states tended to express higher levels of *CCL2* mRNA than those in low EMT states (Fig. 3). Thus, the level of CCL2/*Ccl2* mRNA expression in both human and mouse BC cells correlates with their EMT status.

## Effects of CCL2 on BC cells, mesenchymal stem cells (MSCs), and neutrophils in BC progression

Although the main activity of CCL2 is to recruit CCR2^+^ blood monocytes, studies have reported on its effects on BC cells, MSCs, and neutrophils during BC development and progression.

### Effects of CCL2 on BC cells

Several investigators have examined whether CCL2 directly affects BC cell functions, such as migration, invasion and proliferation, using human BC cells in vitro (Table 3). In a study by Youngs et al., MCF-7 cells migrated in response to CCL2 as well as other chemokines in a dose- and time-dependent manner in a chemotaxis assay, although the responses to each chemokine were variable. ZR-75-1 cells responded to CCL4 and CXCL1 with maximum migration indices of 3.7 and 5.3, respectively, and to CCL4, CXCL8, and CCL2 at lower levels. T47D cells were unresponsive to the chemokines tested, but both MCF-7 and T47D cells bound radiolabeled ligands, suggesting the presence of CCL2 binding sites [63]. Nam et al. found that knockdown of CCL2 in MDA-MB-231 cells reduced their ability to invade in a Matrigel invasion assay and to metastasize to the lung in vivo after injection into the tail vein of combined immune deficient (SCID) mice [45]. Regarding the effect on cancer cell proliferation, Dutta et al. reported that CCL2 did not increase cell proliferation in either CCL2^low^ or CCL2^high^ BC cells but upregulated the phosphorylation of MAP kinase (p42/p44) and increased cell invasion in various BC cells, including MCF-7, MDA-MB-436 and MDA-MB-468 cells. This effect was inhibited by a CCR2 antagonist (Calbiochem CAS 445479-97-0) or a MEK inhibitor (U0126). Knockdown of CCL2 also decreased cell invasion and downregulated the expression of MMP-9 and the key epithelial to mesenchymal transition (EMT) markers N-cadherin and Vimentin [46]. In contrast, Soria et al. noted that CCL2 did not induce EMT in BC cells [35].

**Table 3:** Direct effects of CCL2 on BC cells

Authors	CCL2 effects
Youngs, et al. [63]	Chemotactic response
Nam, et al. [45]	Invasion through Matrigel
Tsuyada et al. [64]	CSC sphere formation and self-renewal
Dutta, et al. [46]	Cell proliferation, cell invasion, MAPK activation, MMP9 expression, EMT marker expression
Fang, et al. [65, 66, 68]	Cell migration, survival, MAPK activation, SMAD3 activation, DCIS progression, regulate cell growth and survival of Luminal B BC cells by inhibiting necrosis and autophagy
Brummer et al. [67]	Drives early BC progression via CCR2
Yao, et al. [69]	BC cell growth, Src activation, PKC activation
Lee, et al. [70]	ERO1-α expression, MMP9 expression, invasion
Li, et al. [38]	Endocrine resistance via PI3K/Akt/mTOR pathway
Chen et al. [72]	Promotes EMT and enhances CSC properties in TNBC via activation of CCL2/AKT/β-catenin signaling
Kanyomse, et al. [73]	Promotes EMT, migration and invasion

Cancer stem cells (CSCs) play critical roles in cancer initiation, progression, and therapeutic resistance. Although many studies have been conducted to characterize the genes and pathways involved in stemness, the factors that regulate CSCs in the tumor microenvironment remain unclear. Tsuyada et al. investigated the effects of stromal fibroblasts on BC stem cells. Compared with normal fibroblasts, primary cancer-associated fibroblasts (CAFs) or fibroblasts activated by cocultured BC cells produced higher levels of CCL2, which stimulated the stem cell-specific sphere-forming phenotype in BC cells and CSC self-renewal. Increased CCL2 expression in activated fibroblasts required signal transducer and activator of transcription (STAT) 3 activation by diverse BC-secreted cytokines and in turn induced NOTCH1 expression and CSC features in BC cells. In a xenograft model of paired fibroblasts and BC cells, loss of CCL2 significantly inhibited tumorigenesis and NOTCH1 expression. In addition, upregulation of both NOTCH1 and CCL2 was associated with poor differentiation in primary BC, further supporting the observation that NOTCH1 is regulated by CCL2. These findings, therefore, suggested that CCL2 represents a potential therapeutic target that can block the cancer-host communication that facilitates CSC-mediated BC progression [64].

Fang et al. reported that in a panel of mouse and human BC cells, CCL2 enhanced cell migration and survival, which was associated with increased phosphorylation of suppressor of mothers against decapentaplegic (SMAD) 3 and p42/44 MAPK proteins. The CCL2 receptor CCR2 was found to be elevated in BC cells, correlating with CCL2 expression. Knockdown of CCR2 expression in BC cells significantly inhibited CCL2-induced migration, survival, and phosphorylation of SMAD3 and p42/44 MAPK proteins. Disruption of SMAD3 expression in BC cells blocked CCL2-induced cell survival and migration and partially reduced p42/44 MAPK phosphorylation. The MEK inhibitor U0126 further reduced cell survival, but not migration, in SMAD3-deficient cells. These findings indicated that SMAD3 signaling through MEK-p42/44MAPK regulated CCL2-induced cell motility and survival, whereas CCL2 induction of MEK-p42/44 MAPK signaling functioned as an alternative mechanism for cell survival independent of SMAD3. Furthermore, CCL2-induced SMAD3 signaling via MEK-p42/44 MAPK regulated the expression and activity of Rho GTPase to mediate CCL2-induced BC cell motility and survival. From these results, the authors suggested that CCL2/CCR2 signaling plays an important role in regulating BC cell motility and survival with implications for the metastatic process [65]. The authors then sought to determine the clinical and functional relevance of the CCL2/CCR2 signaling proteins to DCIS progression. By IHC of DCIS and IDC tissues, the expression of CCL2, CCR2, phospho-SMAD3, and phospho-p42/44 MAPK was shown to be associated with IDC. Using patient-derived xenograft (PDX) models and immortalized hDCIS.01 breast epithelial cells, they showed that breast epithelial cells with high CCR2 and CCL2 levels formed invasive breast lesions that expressed phospho-SMAD3 and phospho-p42/44 MAPK. These studies demonstrated that increased CCL2/CCR2 signaling in breast tissues is associated with DCIS progression and could be a signature for predicting the likelihood of DCIS progression to IDC [66].

Using a mammary intraductal injection (MIND) model to mimic DCIS formation, Brummer et al. explored the role of CCR2 in minimally invasive SUM225 and highly invasive DCIS.com BC cells. CCR2 overexpression increased SUM225 BC cell survival and invasion associated with the accumulation of CCL2-expressing fibroblasts. CCR2-deficient DCIS.com BC cells formed fewer invasive lesions with fewer CCL2^+^ fibroblasts. Cografting CCL2-deficient fibroblasts with DCIS.com BC cells in the subrenal capsule model inhibited tumor invasion and survival associated with decreased expression of the proinvasive factor aldehyde dehydrogenase (ALDH1) and decreased expression of the proapoptotic factor Htra serine protease 2 (HTRA2). Through data mining analysis, high expression of CCR2 and ALDH1 and low HTRA2 expression were correlated with poor prognosis in BC patients. This study demonstrated that CCR2 overexpression in BC drives early-stage BC progression through stromal-dependent expression of CCL2 with important insight into the prognosis and treatment of DCIS [67].

Luminal A and B BCs are the most prevalent types of BC diagnosed in women. Compared to patients with the luminal A type, patients with the luminal B type show increased disease recurrence and shorter overall survival. The mechanisms that regulate the luminal B subtype are not well understood. Fang et al. [68] aimed to determine the role of CCL2 expression in luminal B BC cells. CCL2 expression was significantly increased in luminal B breast tumors and tumors in MMTV-PyMT or MMTV-Neu mice compared to normal breast tissue or luminal A breast tumors. Silencing CCL2 expression by TAT cell-penetrating peptides noncovalently cross-linked to siRNAs (Ca-TAT/siRNA) significantly reduced CCL2 expression in PyMT mammary tumors and decreased cell proliferation and survival. CCL2 silencing in PyMT carcinoma cells or BT474 luminal B BC cells also decreased cell growth and viability with increased necrosis and autophagy. These findings indicated that CCL2 expression is increased in luminal B BC cells and is important for the regulation of cell growth and survival by inhibiting necrosis and autophagy.

The effects of CCL2 on BC cells were also demonstrated using other in vivo models. Yao et al. used the human MCF10CA1d xenograft model (basal-like BC) and found that the growth of primary tumors was significantly increased by cotransplantation of fibroblasts derived from BC patients expressing high levels of CCL2, which was inhibited by mutation of the *CCL2* gene in fibroblasts by CRISPR‒Cas9 technology. CRISPR‒Cas9 gene mutation of *CCR2* in MCF10CA1d cells inhibited tumor growth and TAM recruitment, which was also observed by *Ccr2* shRNA knockdown in the mouse 4T1 TNBC model. Reversed-phase protein array analysis revealed that cell-cycle protein expression was associated with CCR2 expression in basal-like BC cells, such as BT-20 and HCC1937. CCL2 treatment of basal-like BC cell lines increased proliferation and cell cycle progression associated with Src and protein kinase C (PKC) activation. Pharmacological inhibition of Src and PKC showed that Src and PKC negatively regulated the expression of the cell cycle inhibitor protein p27KIP1 and were necessary for CCL2-induced BC cell proliferation. These results suggested that CCL2/CCR2 chemokine signaling is a mitogenic pathway and a cell cycle regulator in BC cells [69].

Lee et al. investigated the effects of TAMs on the phenotypic changes in nonneoplastic MCF10A human breast epithelial cells using human monocytic THP-1 cells treated with phorbol 12-myristate 13-acetate as TAMs. Coculture of MCF10A cells with TAMs induced EMT, an invasive phenotype, and MMP-9 upregulation. Comparative proteomic analysis revealed that endoplasmic reticulum oxidoreductase (ERO)1-α was increased in MCF10A cells cocultured with TAMs. ERO1-α was crucial for the TAM-induced invasive phenotype and MMP-9 upregulation involving the transcription factors c-Fos and c-Jun. Cytokine array analysis showed that the levels of IL-6, CXCL1, CCL2, CXCL8, and granulocyte-macrophage colony-stimulating factor (GM-CSF)/CSF2 were increased in the conditioned media of cocultured cells. CCL2 was secreted from TAMs and led to the upregulation of ERO1-α, MMP-9, and invasiveness in MCF10A cells [70].

TAMs are involved in the resistance of prostate cancer patients to androgen blockade therapy [71]. Li et al. aimed to elucidate the relationship between TAMs and the endocrine-resistant phenotype of BC and found a correlation between TAMs and tamoxifen resistance in BC patients. CCL2 secreted by TAMs activated the phosphoinositide 3 kinase (PI3K)/Akt/mammalian target of rapamycin (mTOR) signaling pathway in MCF-7 and T47D cells and promoted an endocrine resistance feedback loop in the TME [38].

Chen et al. explored the mechanism by which TAMs regulate EMT and CSC properties in TNBC. They demonstrated a high infiltration of TAMs into TNBC tissues. TAMs, polarized into the M2-like type, promoted EMT and CSC properties in TNBC cells, such as BT549 and HCC1937 cells. CCL2 secreted by TAMs activated Akt signaling, which in turn increased the expression and nuclear localization of β-catenin, providing a novel mechanism by which TAMs promote EMT and enhance CSC properties in TNBC via activation of CCL2/AKT/β-catenin signaling [72]. Kanyomse et al. [73] also reported that CCL2 promoted EMT, migration, and invasion of BC cells using BT-549 and MDA-MB-231 cells.

### Effects of CCL2 on bone marrow-derived mesenchymal stem cells (MSCs)

MSCs are a subset of nonhematopoietic stem cells that reside in the bone marrow stroma and are capable of self-renewal and differentiation into cells of connective tissue lineages. MSCs contribute to the maintenance and regeneration of connective tissues through engraftment induced by tissue-produced factors when new connective tissue cells are needed during wound healing or scar formation processes [74]. Cancer cells also require the formation of connective tissues to support their growth and progression. This led to studies investigating the homing of MSCs to tumors to determine whether they can be used as a targeted delivery vehicle for therapies. Dwyer et al. reported that MSCs migrated in response to recombinant human CCL2 in a dose-dependent manner in a transwell migration assay. Furthermore, the addition of an anti-CCL2 antibody significantly reduced the migration of MSCs in response to the conditioned medium of ex vivo cultured tumors containing CCL2. These results suggested that tumor-derived CCL2 may be responsible for the recruitment of MSCs into BC tissues [47].

### Effects of CCL2 on tumor-entrained neutrophils (TENs)

The role of neutrophils in cancer cell metastasis has been studied by many investigators, and the results of those studies have indicated that they play both antitumor and protumor roles [75]. Granot et al. found that in a mouse 4T1 BC model, the accumulation of TENs (neutrophils induced by the primary tumor) in the lung during premetastatic stages limited metastatic progression via neutrophil-derived H_2_O_2_, which provided antitumor functions, and tumor cell-derived CCL2 was responsible for the accumulation of TENs [76].

Lavender et al. evaluated whether CCL2 can “entrain” naïve neutrophils to enhance tumor cell killing using three different mouse BC models, 4T1, 67NR, and PyMT, with different aggressiveness: 4T1 and PyMT cells as metastatic cells and 67NR cells as nonmetastatic cells. All three BC cell types produced similar amounts of CCL2. Neutrophils were isolated by washing the peritoneal cavity of naïve (naïve neutrophils) or tumor-bearing mice (TENs). The addition of exogenous CCL2 to cocultures of BC cells and neutrophils enhanced the ability of TENs to kill the less aggressive 67NR variant of 4T1 BC cells. However, exogenous CCL2 did not enhance the killing of more aggressive 4T1 or PyMT BC cells by either naïve neutrophils or TENs. Antitumor activity was also not observed in vivo. Intranasal delivery of CCL2 to BALB/c mice markedly enhanced the seeding and outgrowth of 67NR cells in the lung and increased the recruitment of CD4^+^ T cells and CD8^+^ central memory T cells into the lungs of tumor-bearing mice. There was no significant increase in the recruitment of CD19^+^ B cells or F4/80^+^, Ly6G^+^, and CD11c^+^ myeloid cells. CCL2 had an equal effect on the recruitment of CD206^+^ (pro-tumor) and MHC II^+^ (antitumor) populations of macrophages, thus balancing the pro- and antitumor macrophage cell populations. These findings suggested that CCL2 may have a more protumor effect than an antitumor effect [77]. The CCL2 effects introduced in this section are summarized in Table 3.

## Roles of CCL2 in the development and progression of BC in mouse BC models

### Critical roles of CCL2 in macrophage recruitment and lung metastasis in BC

As described above, when CCL2 was first purified, the role of TAMs was still controversial [18]. Although the protumor activity of TAMs had been strongly suggested [78], several years of investigation were needed to experimentally clarify their role. Colony-stimulating factor-1 (CSF-1), also known as macrophage colony-stimulating factor (M-CSF), is the major growth factor for the mononuclear phagocytic lineage [79], and overexpression of CSF-1 and CSF-1R was found in a large percentage of BC cases in which its expression was correlated with poor prognosis [80]. To determine the role of CSF-1 in BC development, Lin et al. [81] crossed *Csf-1*-null (*Csf1*^*op*^*/Csf1*^*op*^) mice with mammary cancer–susceptible MMTV-PyMT mice [82]. In the absence of CSF-1, the formation and growth of primary mammary tumors were not altered, but the recruitment of macrophages and both the progression to malignant forms and lung metastasis of tumor cells were significantly delayed. Furthermore, transgenic expression of *Csf-1* specifically in the mammary epithelium of *Csf-1* null mice and wild-type (WT) mice accelerated tumor progression and lung metastasis. This study established the protumor activity of CSF-1-dependent macrophages in BC and perhaps other cancers.

To define the contribution of CSF-1 to the generation of a macrophage population that promotes metastasis in this BC model, the same investigators used in vitro cultured tumor cells derived from mammary tumors of MMTV-PyMT mice, including primary tumor cells and highly metastatic Met-1 cells [83]. In the primary tumor that developed after tumor cell transplantation, CD11b^+^Gr1^-^Ly6C^-^ resident monocytes were preferentially recruited, whereas CD11b^+^Gr1^+^Ly6C^+^CCR2^+^ inflammatory monocytes (IMs) were preferentially recruited to pulmonary metastases. These inflammatory monocytes differentiated into CD11b^+^Gr1^-^ metastasis-associated macrophages (MAMs) and were involved in the extravasation and growth of BC cells at the metastatic site [84].

The mechanisms of IM recruitment to pulmonary metastases were subsequently determined using multiple mouse models. In the models in which human MDA-MB-231 cells were orthotopically injected or MDA-MB-231-derived 4173 cells were injected via the tail vein into SCID mice, either anti-human CCL2 antibody (Ab) or anti-mouse CCL2 Ab significantly inhibited lung metastasis, suggesting that the infiltration of IMs was dependent on CCL2 synthesized by both tumor and stromal cells. IMs then enhanced the extravasation of tumor cells by a mechanism that required monocyte-derived VEGF. The results of an Oncomine search indicated that CCL2 was overexpressed in both the tumor and tumor stroma of invasive human BCs and correlated with the development of metastatic disease and poor prognosis [85]. A series of studies by this group established an important role for TAMs and CCL2 in progression of BC, especially lung metastasis. The researchers continued their study and showed that MAMs that differentiated from IMs expressed higher levels of CCR1 than IMs. CCL2 increased the expression level of the CCR1 ligand CCL3 in MAMs via CCR2, and the signaling generated by the CCL3/CCR1 interaction enhanced and stabilized the cancer cell-MAM interaction in part through integrin α4 binding to VCAM1 expressed on tumor cells in an autocrine manner. Thus, there is a signaling relay from CCL2/CCR2 to CCL3/CCR1 during the process of BC lung metastasis, and blocking the distal part of this signaling relay may have more impact on metastatic disease than blocking upstream targets because of likely lower toxicity [86, 87] (Table 4).

**Table 4:** Effects of the disruption of the CCL2/CCR2 axis on BC progression in mouse BC models

Authors [Ref]	BC models	Treatments or knockout	Phenotypical changes	
			Primary tumor size	Lung metastasis
Qian et al. [85]	Met-1	Anti-human or mouse CCL2 Ab, KD	N/A	Decreased
	4341 (MDA-MB-231-derived)	Anti-human or mouse CCL2 Ab, KD	N/A	Decreased
	MDA-MB-231	Anti-human or mouse CCL2 Ab	No change	Decreased
Fujimoto et al. [34]	MDA-MB-231	Anti-mouse Ab	Decreased	Decreased
Yao et al. [89]	MCF10CA1d	Anti-human CCL2 Ab	No change	No change
Li et al. [38]	AT-3 (MMTV-PyMT-derived)	*Ccl2*^-/-^ mice	Growth delayed	Increased
		*Ccr2*^-/-^ mice	Growth delayed	Increased
	AT-3 and 4T1	Anti-CCL2 Ab	Increased and then reduced	Decreased
	MMTV-PyMT mice	*Ccl2*^-/-^ mice	Increased and then reduced	Increased
		*Ccr2*^-/-^ mice	Increased and then reduced	Increased
Brummer et al. [97]	MMTV-PyMT cells, intraductally injected	*Ccr2* KD	Reduced growth and invasion	N/A
Chen et al. [100]	MMTV-neu/HER2 mice	*Ccl2*^-/-^ mice	Growth slower	N/A
		*Ccr2*^-/-^ mice	Growth increased	N/A
		CCR2 antagonist	Decreased	N/A
Yoshimura et al. [51] and here	4T1	*Ccl2*^-/-^ mice	No change	Decreased
		*Ccr2*^-/-^ mice	Decreased	No change
Bonapace et al. [106]	4T1, 4T1.2, J110, Met-1	Anti-mouse CCL2 Ab	No change	Decreased
		Discontinuation of Ab	N/A	Increased
Svensson et al. [153]	MMTV-PyMT mice, MCF-7	Anti-human CCL2 + Anti-human CCL5 Abs	Decreased	N/A
Yumimoto et al. [142]	EO771	CCR2 antagonist	N/A	Decreased
Linde et al. [91]	MMTV-PyMT	CCR2 antagonist	N/A	Decreased infiltration of intra-epithalial macrophages
Rogic et al. [108]	A3250 (inflammatory BC cells)	*Ccl2* KD	Decreased	Decreased
Kim et al. [53]	T11, EO771, PyMT-M, AT-3, 2208 L, PyMT-N	*Ccr2*^*-/-*^ *mice*	Heterogenous	N/A

*KD* knockdown. *N/A* not available

Fujimoto et al. used the same MDA-MB-231 mouse BC transplantation model and evaluated the effect of the neutralization of mouse CCL2 on macrophage infiltration and tumor development. A smaller number of macrophages were observed in the tumors of mice treated with anti-mouse CCL2 polyclonal goat Ab (R&D Systems) compared with control mice. The tumor volume of control mice was 1.5 times larger than that of anti-mouse CCL2 Ab-treated mice. Furthermore, microvessel density, defined with anti-CD31 Ab staining, was reduced in mice treated with anti-mouse CCL2 Ab, suggesting that CCL2 produced by stromal cells, but not cancer cells, plays a critical role in tumor growth and angiogenesis during disease progression in this BC model [34]. Both Qian et al. and Fujimoto et al. used the same xenograft model with human MDA-MB-231 cells, but their findings were slightly different.

Yao et al. implanted osmotic pumps containing control IgG or anti-human CCL2 neutralizing antibody (R&D system, MAB279) in nude mice bearing MCF10CA1d breast tumor xenografts and analyzed CCL2 levels and tumor progression over 4 weeks. The cell line MCF10CA1d was derived from MCF10AneoT cells that were transfected with T24 Ha-ras and a model for basal-like BC [88]. Although the antibody inhibited the migration of MCF10CA1d cells in response to CCL2 in vitro, it did not significantly affect tumor growth, invasion, macrophage recruitment, or tumor angiogenesis. The antibody did not affect murine CCL2 levels but significantly increased human CCL2 levels in the plasma or tumor interstitial fluid. Since the antibody effectively reduced CCL2 levels in cultured cells, such as fibroblasts and BC cells and CCL2 levels were restored once the antibody was removed, the results obtained from in vivo experiments were puzzling. The authors concluded that there are limitations to the use of CCL2-neutralizing antibodies as therapeutic agents [89].

Linde et al. examined a potential role of macrophages in the process of cancer cell dissemination during evolutionary early stages of BC progression using MMTV-HER2 mice. Previous studies with BC patients and spontaneous mouse tumor models showed that cancer cell dissemination occurs during early stages of cancer when abnormal lesions are diagnosed by light microscopy as premalignant or preinvasive [90]. However, the mechanism of early dissemination remains unclear. In MMTV-HER2 mice, a mouse HER2^+^ BC model, a previously described HER2^+^/P-p38^lo^/P-ATF2^lo^/ TWIST^hi^/E-cadherin^lo^ subpopulation of early-evolved cancer cells required macrophages for early dissemination. Depletion of macrophages specifically during premalignant stages reduced early dissemination and resulted in reduced metastatic burden at end stages of cancer progression. Treatment of MMTV-HER2 mice carrying only early lesions with a CCR2 antagonist (RS504393) reduced the number of intraepithelial macrophages. Mechanistically, in premalignant lesions, CCL2 produced by cancer cells and myeloid cells attracted CD206^+^/Tie2^+^ macrophages and induced Wnt-1 upregulation, which in turn downregulated E-cadherin junctions in HER2^+^ early cancer cells. Macrophage-containing tumor microenvironments of metastasis were generated in the premalignant lesions and could operate as portals for intravasation. These data supported a causal role for macrophages in early dissemination that affects long-term metastatic development much later in cancer progression. A pilot analysis of human specimens revealed the presence of intraepithelial macrophages and loss of E-cadherin junctions in DCIS, supporting the potential clinical relevance of their findings [91]. Although the effect of the CCR2 antagonist on lung metastasis of BC cells was not presented, it is presumed that blocking CCR2 interferes with BC lung metastasis by inhibiting the recruitment of intraepithelial macrophages.

### Effects of CCL2 or CCR2 deficiency on the development and progression of BC

Mice deficient in CCL2 [92, 93] or CCR2 [94, 95] were generated to define the in vivo role of CCL2 in various disease models. These mice have been used to determine the contribution of CCL2/CCR2 signals to the progression of BC in combination with BC-susceptible transgenic mouse models or transplantable mouse BC models.

#### MMTV-PyMT model

Li et al. first injected AT-3 cells (derived from tumors of MMTV-PyMT mice [54]) into WT, *Ccl2*^-/-^ or *Ccr2*^-/-^ mice. Although primary tumors developed in all three mouse strains, the growth of the primary tumor was significantly delayed in *Ccl2*^-/-^ and *Ccr2*^-/-^ mice compared with WT mice. Spontaneous pulmonary metastasis was significantly increased in both *Ccl2*^-/-^ and *Ccr2*^-/-^ mice. Splenomegaly was also reduced in *Ccl2*^-/-^ mice and *Ccr2*^-/-^ mice, likely reflecting the smaller tumor size in those mice. The proportions of CD11b^+^ and CD11b^+^Gr1^+^ cells (defined as MDSCs) in the primary tumor were decreased in both *Ccl2*^-/-^ and *Ccr2*^-/-^ mice, but there were no significant differences in the ratio of CD4^+^, CD8^+^, or γδ T cells, B cells, CD11c^+^ cells, natural killer (NK), or NKT cells observed. Thus, the loss of CCL2 or CCR2 in the host resulted in decreased MDSC recruitment and tumor growth but increased lung metastases.

The authors next used anti-mouse CCL2 monoclonal antibody (C1142, Janssen R&D). Neutralization of CCL2 significantly inhibited the growth of primary AT-3 tumors 42 days after implantation and 4T1 tumors 29 days after implantation. Tumor volumes increased in an early phase in response to anti-CCL2 Ab treatment but rapidly reverted over time and resulted in the inhibition of primary tumor growth, suggesting that CCL2 neutralization may act in a biphasic manner. There was no obvious increase in the abundance of tumor-infiltrating leukocytes (TILs). Surprisingly, unlike in *Ccl2*^-/-^ or *Ccr2*^-/-^ mice, there was a significant decrease in the number of spontaneous lung metastases in mice bearing either AT-3 or 4T1 tumors in response to Ab treatment. This difference might be due to the timing of the CCL2 intervention and/or the effectiveness of CCL2 neutralization. It was also suggested that the administration of anti-CCL2 Ab during the tumor induction phase is detrimental, whereas Ab treatment is inhibitory after the tumor induction phase or when tumors are already established.

Finally, the authors crossed either *Ccl2*^-/-^ [92] or *Ccr2*^-/-95^ mice with MMTV-PyMT mice [82] and monitored the development of tumors. The development of palpable tumors was accelerated in *Ccl2*^-/-^ or *Ccr2*^-/-^ mice; however, tumors in *Ccl2*^-/-^ or *Ccr2*^-/-^ mice did not grow aggressively but rather grew at a significantly reduced growth rate. There was a significant increase in lung metastases in both *Ccl2*^-/-^ and *Ccr2*^-/-^ mice compared with control mice. Thus, the CCL2/CCR2 axis appeared to play a dual role by promoting early tumor development (pro-tumor) but sustaining the growth and lung metastasis of BC cells (antitumor) [96].

Instead of using *Ccr2*^-/-^ mice, Brummer et al. knocked down CCR2 expression in mammary tumors that developed after intraductal injection of MMTV-PyMT mammary carcinoma cells with siRNAs complexed to TAT cell penetrating peptides by calcium cross-linking. Selective targeting of CCR2 inhibited tumor growth and invasion, elevated the infiltration of CD8^+^ T cells, decreased the infiltration of M2 macrophages and decreased angiogenesis. Coculture experiments demonstrated that these stromal cell responses were mediated by tumor-derived CCL2 and CCR2-mediated suppression of the T-cell activating cytokine CD154 (CD40L). Coculture studies also indicated that CCR2-induced stromal cell recruitment was important for tumor cell proliferation and invasion. In human breast tumor tissues, CD154 expression inversely correlated with CCR2 expression and correlated with relapse-free survival. These results suggested a role for epithelial CCL2/CCR2 signaling to regulate mammary tumor growth, invasion and inflammation via suppression of CD154 signaling  [97].

#### MMTV-neu (MMTV-HER2) model

Chen et al. crossed *Ccl2*^-/-^ mice [92] or *Ccr2*^-/-95^ mice with mice from another transgenic line, namely, MMTV-neu mice (neu^+^) [98], and examined the development of breast tumors that spontaneously arose. *Ccl2*^-/-^ neu^+^ mice survived significantly longer than neu^+^ mice. Tumor-free survival was unchanged in *Ccl2*^-/-^ neu^+^ mice compared to neu^+^ mice, but the rate of tumor growth, measured as total tumor burden or as the rate of growth of the single largest tumor mass, was slower in the absence of CCL2. In contrast, loss of CCR2 significantly shortened survival with earlier appearance and more rapid growth of tumors. Blocking CCR2 with CCX872, a CCR2 antagonist, suppressed tumor growth without extending tumor-free survival and prolonged overall survival in neu^+^ mice. Thus, the effects of CCX872 were similar to those of CCL2 deficiency but different from those of CCR2 deficiency. This finding suggested that the CCL2/CCR2 axis promotes tumor growth in the MMTV-neu model and that genetic disruption of the *CCR2* gene promotes tumor growth through different pathways.

The proportion of circulating monocytes was the same in tumor-free WT, *Ccl2*^-/-^, or *Ccr2*^-/-^ mice. Interestingly, the number of circulating monocytes was reduced in tumor-bearing mice. Disruption of the *Ccl2* or *Ccr2* gene specifically reduced the proportion of Ly6C^hi^ monocytes. The gene expression profile showed a more profound alteration in *Ccr2*^-/-^ monocytes than in *Ccl2*^-/-^ monocytes compared to WT monocytes. *Ccl2*^-/-^ monocytes showed significant differences in the expression of 808 genes compared to WT monocytes. Although the expression of 766 genes among the 808 genes was also altered in *Ccr2*^-/-^ monocytes, *Ccr2*^-/-^ monocytes showed significant changes in an additional 1621 genes. To explore the implications of these alterations, the authors examined the expression levels of specific genes that are markers for Ly6C^hi^ and Ly6C^lo^ monocytes [99]. *Ccl2*^-/-^ monocytes showed a bias toward the Ly6C^lo^ signature, including reduced expression of *Mmp8*, *Irf4*, *Msr1* and *Klf4* and augmented expression of *Tgfbr3*, *Mr1*, *Runx3* and *Ets1*. *Ccr2*^-/-^ monocytes were markedly deficient in pathways related to host defense, such as wounding, inflammation, and leukocyte locomotion.

CCL2 did not directly affect tumor cell proliferation because tumor cells isolated from neu^+^, *Ccl2*^-/-^ neu^+^, and *Ccr2*^-/-^ neu^+^ mice grew at similar rates in vitro. The addition of neutralizing anti-CCL2 Ab (R&D Systems) to WT tumor cells or exogenous CCL2 to *Ccl2*^-/-^ tumor cells did not affect their proliferation. Neutralization of CCL2 did not induce apoptosis or inhibit proliferation of human BC cells, including MDA-MB-231 cells [100].

Since angiogenesis in tumor tissues can be achieved, at least in part, by the recruitment of endothelial progenitor cells (EPCs) [101], the authors examined whether the presence of mammary carcinomas affects the development of CD45^-^CD117/c-Kit^+^Flk1/Vegfr2^+^ EPCs and whether CCL2 and CCR2 play a role in the process. The presence of tumors in WT mice increased the proportion of EPCs in peripheral blood by twofold. Deletion of *Ccr2* increased the proportion of circulating EPCs in WT mice, and the presence of mammary tumors further increased this proportion by twofold. However, the number of EPCs in bone marrow was not significantly increased, suggesting that mammary tumors increase EPC mobilization without enhancing EPC production. In contrast to *Ccr2* deletion, deletion of *Ccl2* reduced circulating EPCs by 50% and bone marrow EPCs by 80%, and neither proportion increased in the presence of tumors. This finding suggested that CCL2 is required for the development of EPCs and their mobilization by mammary tumors. Tumors in *Ccl2*^-/-^ mice contained the same total number of TAMs as tumors in WT mice, but *Ccl2* disruption was associated with a marked reduction in the numbers of EPCs in the bone marrow and circulation, which may suppress tumor angiogenesis [100].

#### Transplantable 4T1 BC model

As described above, positive staining for CCL2 was detected in both parenchymal and stromal components of human BC tissues by IHC; however, stromal cell-derived CCL2 was suggested to be functionally important in recruiting TAMs (Table 1). We aimed to determine whether CCL2 produced by nontumor stromal cells affects the growth and lung metastasis of BC cells by transplanting 4T1 cells into the mammary pad of WT or *Ccl2*^-/-^ mice [51, 93]. The 4T1 cell line is a murine TNBC cell line derived from a mammary tumor that spontaneously developed in a BALB/c mouse foster-nursed by a C3H female mouse (BALB/cfC3H) [57, 102]. In female BALB/c mice, 4T1 cells implanted at the orthotopic mammary fat pad spontaneously metastasize to multiple organs, including the bone, lung, and liver [103]. As already noted above, 4T1 cells are a mixture of subclones with different gene expression profiles and metastatic potentials [61, 62]. Thus, 4T1 cells are an excellent model for exploring the mechanisms of TNBC progression and metastasis and, as has been noted above, have been used in many studies.

In vitro, 4T1 cells spontaneously produced a low level of CCL2, which could be increased in response to the TLR4 ligand lipopolysaccharide (LPS) or TNFα [51]. Primary tumors at the injected site grew similarly in both WT and *Ccl2*^-/-^ mice; however, lung metastases were markedly reduced in *Ccl2*^-/-^ mice, with significantly longer mouse survival. In tumor-bearing WT mice, the serum CCL2 concentration markedly increased 1 week after inoculation of 4T1 cells, peaked at 2 weeks, and decreased thereafter despite the primary tumor continuing to grow. In contrast, serum CCL2 levels were undetectable in tumor-bearing *Ccl2*^-/-^ mice, and the level of *Ccl2* mRNA in tumors of *Ccl2*^-/-^ mice was markedly lower. These results indicated that stromal cells, but not cancer cells, were the major cellular source of CCL2 in 4T1 tumors. Transplantation of *Ccl2*^-/-^ bone marrow cells into WT mice did not alter the incidence of lung metastasis, whereas transplantation of WT bone marrow cells into *Ccl2*^-/-^ mice increased lung metastasis, suggesting that CCL2 produced by both myeloid cells and nonmyeloid cells was important. The primary tumors of *Ccl2*^-/-^ mice consistently developed necrosis earlier than those of WT mice and showed decreased macrophage infiltration and reduced angiogenesis as defined by positive F4/80 and CD31 staining, respectively. Interestingly, 4T1 cells that metastasized to the lung constitutively expressed higher levels of CCL2 than the original 4T1 cells, and intravenous injection of 4T1 cells producing a higher level of CCL2 resulted in increased numbers of tumor foci in the lungs of WT and *Ccl2*^-/-^ mice. These results indicated that stromal cell-derived CCL2 in the primary tumor promotes lung metastasis of 4T1 cells but that tumor cell-derived CCL2 can also contribute once tumor cells enter the circulation [51].

Gu et al. injected luciferase-labeled 4T1 cells into the mammary pad of WT and *Ccl2*^-/-^ mice (no information on the source of *Ccl2*^-/-^ mice) and compared the growth of tumors and lung metastasis. The absence of CCL2 in the host reduced the tumor weight by more than 4-fold, the number of lung metastases, and the proportion of MDSCs (CD45^+^/CD11b^+^/Gr1^+^) in both tumors and metastases, suggesting that the expression of CCL2 in the host could promote the proliferation and metastasis of BC via the recruitment of MDSCs [104]. Although the exact reason why the growth of primary tumors was markedly reduced in the absence of CCL2 in this study is not clear, the use of luciferase-labeled 4T1 cells may have caused this difference.

**Fig. 4:**
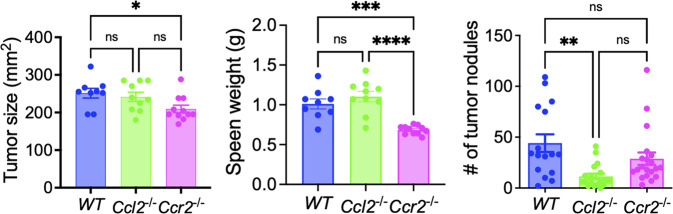
Comparison of the primary tumor size, spleen weight, and lung metastases after orthotopic implantation of 4T1 breast cancer cells in WT, *Ccl2*^-/-^ or *Ccr2*^-/-^ mice. One hundred thousand 4T1 cells were injected into the mammary pad of mice of each genotype, and the mice were euthanized 4 weeks after tumor cell inoculation

We also transplanted 4T1 cells into the mammary pad of *Ccr2*^-/-^ mice [94]. The size of tumors and the weight of spleens were significantly lower in *Ccr2*^-/-^ mice than in WT mice, but there was no significant difference in the number of lung metastases between WT and *Ccr2*^-/-^ mice (Fig. 4). Thus, in agreement with the studies by Li et al. and Chen et al., the progression of BC in *Ccl2*^-/-^ mice appears different from that in *Ccr2*^-/-^ mice.

Kim et al. orthotopically injected 6 types of mouse TNBC cells, namely, T11, EO771, PyMT-M, AT-3, 2208 L, and PyMT-N, into *Ccr2*^-/-^ mice [53, 95]. Tumors of T11, EO771, and PyMT-M were macrophage-enriched subtype (MES) tumors, whereas tumors of AT-3, 2208L, and PyMT-N were neutrophil-enriched subtype (NES) tumors. Infiltration of Ly6C^high^ monocytes was reduced by 3- to 7-fold in all models; however, a significant reduction (more than 2-fold change) in tumor-infiltrating macrophages (TIMs) (CD11b^+^Ly6G^−^Ly6C^−^F4/80^+^) was observed only in MES tumors, indicating that CCR2 dependency was MES specific. In contrast, TIMs in NES tumors were not affected by CCR2 deficiency. The impact of CCR2 deficiency on tumor growth, T-cell infiltration, proliferation, apoptosis, or angiogenesis was heterogeneous. The results of this study may partly explain the different outcomes reported by different laboratories using *Ccl2*^-/-^ or *Ccr2*^-/-^ mice. Additional studies are necessary to identify the mechanisms leading to the phenotypic differences observed between *Ccl2*^-/-^ and *Ccr2*^-/-^ mice.

### Aggravated lung metastasis by anti-CCL2 Ab withdrawal

Bonapace et al. investigated the effect of CCL2 neutralization on tumor growth and metastasis in syngeneic mouse transplantation models, including 4T1, 4T1.2 (BALB/c), and J110 (derived from FVB-MMTV-AIB1 mouse tumor [105] and Met-1 cells [83]), producing similar levels of CCL2. Although anti-CCL2 Ab (NA/LE hamster anti-mouse CCL2, clone 2H5) treatment had no effect on primary tumor growth, it reduced the number of lung metastases and circulating tumor cells (CTCs). Intravital imaging showed that Ab treatment reduced cancer cell motility and blood vessel leakiness in the tumor, and more pericytes were found around blood vessels. Decreased blood vessel leakiness by Ab treatment correlated with fewer CTCs and reduced numbers of intratumoral macrophages. Therefore, CCL2 neutralization limits metastases not only through its effects on premetastatic niches but also by limiting cancer cell intravasation at the primary site [106].

Next, they examined the persistence of the anti-metastatic effect after discontinuation of Ab treatment. The Ab was cleared within 10 days after the discontinuation of treatment, and the CCL2 concentration rebounded in the lungs. Surprisingly, discontinuation of Ab treatment accelerated mouse death. Ten days after the interruption of Ab treatment, there was a dramatic increase in the number of lung and liver metastases and an increase in CTC numbers. Thus, although Ab treatment reduced metastases, interruption of the treatment aggravated metastasis. The antimetastatic effect persisted when mice were treated continuously with anti-CCL2 antibody. The exacerbated lung metastasis and death after the interruption of antibody treatment was the result of monocyte release from the bone marrow and enhancement of cancer cell mobilization from the primary tumor as well as blood vessel formation and increased proliferation of metastatic cells in the lungs in an IL-6- and VEGF-A-dependent manner. Neutralization of CCL2 and IL-6 markedly reduced metastases and increased survival of the animals. This study called for caution when considering CCL2 inhibitors as a single therapy in metastatic diseases and highlighted the tumor microenvironment as a critical determinant of successful antimetastasis therapy [106].

### Effects of CCL2 overexpression on BC development

To investigate the role of CCL2 in healthy breast development and the effect of increased CCL2 production by mammary epithelial cells on the risk of cancer initiation, Sun et al. developed a transgenic mouse model, *Mmtv-Ccl2*, in which CCL2 is constitutively expressed by the mammary epithelium. Compared to control mice, mice with overexpression of *Ccl2* in the mammary epithelium exhibited an increased number of macrophages, increased density of stroma and collagen and elevated mRNA levels of the encoding matrix remodeling enzymes lysyl oxidase (LOX) and tissue inhibitor of matrix metalloproteinase (TIMP) 3. Transgenic mice also exhibited increased susceptibility to 7,12-dimethylbenz(a)anthracene (DMBA)-induced mammary tumors. In a paired sample cohort of human breast tissue, the abundance of epithelial-cell-associated CCL2 was higher in breast tissue of high mammographic density than in tissue of low mammographic density. Thus, constitutive CCL2 production by mouse mammary epithelial cells results in the induction of low-level chronic inflammation that increases stromal density and elevates the risk of BC [107].

Gu et al. overexpressed CCL2 only in the mouse lung by inhalation of adeno-associated virus (AAV) and examined whether CCL2-mediated lung metastasis of BC cells is due to the production of CCL2 in the target organ rather than an effect from the primary tumor. After 4 weeks of AAV inhalation (one inhalation dose every week), the expression of CCL2 in the lung significantly increased. 4T1 cells were transplanted into the mammary pad of the mice 7 days after the first AAV inhalation. Overexpression of CCL2 in lung epithelial cells dramatically increased the number of metastatic foci without affecting the growth of the primary tumor and MDSC infiltration in the lung but not in the primary tumor, leading to the conclusion that high expression of CCL2 in the lung was associated with MDSC recruitment and contributed to the establishment of a microenvironment that promoted lung metastasis of BC cells [104]. Thus, increased CCL2 production by mammary epithelial cells and cells in remote organs promoted the development and progression of BC.

### Role of CCL2 in inflammatory breast cancer (IBC)

IBC is a highly aggressive type of BC with distinct clinical and histopathological features, but its molecular mechanism remains unclear. Rogic et al. reported that a human IBC cell line, A3250, recapitulated key features of IBC in a mouse xenograft model, including skin erythema, diffuse tumor growth, dermal lymphatic invasion, and extensive metastasis. A3250 cells expressed high levels of CCL2 and formed tumors that were enriched in macrophages. CCL2 knockdown in cancer cells led to a marked reduction in macrophage infiltration, tumor cell proliferation, skin erythema, and metastasis. Furthermore, primary human IBC tissues showed dense macrophage infiltration and a macrophage-enriched RNA expression signature. These results indicated that IBC cell-derived CCL2 is a key factor driving increased macrophage infiltration and indirect tumor growth by activating multiple inflammatory pathways [108]. Tarek et al. also reported that ex vivo patient-derived explants of IBC were characterized by high-level secretion of CCL2 along with IL-6 [109].

### Role of CCL2 in bone metastasis

In advanced BC cases, bones, as well as lungs, are well-known target organs for metastasis, and nearly 80% of advanced BC patients suffer from bone metastasis [110, 111]. In addition, because of bone’s specific structure and its low blood flow, it is difficult to surgically remove metastatic tumors or effectively deliver anticancer drugs, making complete remission almost impossible to achieve [112]. To determine the mechanisms underlying the spread of BC cells to bone, Takahashi et al. established two mouse 4T1 BC-derived cell lines, 4T1E and 4T1E/M3. 4T1E cells were obtained after transfection of 4T1 cells with the neomycin resistance gene and selection using G418. 4T1E cells were then intravenously injected into BALB/c mice, and metastasized cells were harvested from bone marrow. After repeating this process three times, 4T1E/M3 cells were established [113]. Gene and protein expression in 4T1E and 4T1E/M3 cells was then analyzed. The production of CCL2 was dramatically reduced in 4T1E/M3 cells, and restoration of CCL2 expression in 4T1E/M3 cells diminished their metastasis to the bone and the lung. Overexpression of CCL2 in 4T1E/M3 cells significantly reduced not only in vitro anchorage-independent cell growth and cell migration but also mRNA and cell surface expression of ICAM-1. In contrast, knockdown of CCL2 in 4T1E cells augmented their metastatic spread to the bone (spine) and the lung. The expression of ICAM-1 was also upregulated in 4T1E-derived CCL2 knockdown cells. These results suggested that endogenous CCL2 production by BC cells may negatively regulate their metastasis to the bone and the lung in their model and that expression of ICAM-1 plays a crucial role in that process [112]. Thus, the role of CCL2 identified in this study was the opposite of that identified for lung metastasis. In this study, 4T1 cells were transfected with the *neomycin* gene construct, and several rounds of selection/cloning were employed, potentially contributing to the different outcomes. It would also be interesting to orthotopically inject 4T1E cells into mice and examine their growth and metastasis to bone.

### Role of CCL2 in brain metastasis

The brain is another target organ of BC metastasis, and brain metastasis occurs in approximately 15% of women with newly diagnosed metastatic breast cancer. Conventional therapies for BC brain metastases are largely ineffective because of BC cell chemoresistance and impermeability of the blood‒brain barrier. A better understanding of the mechanisms that allow BC cells to metastasize to the brain is necessary to effectively prevent or treat BC brain metastasis. Similar to its role in BC lung metastasis, CCL2 was found to facilitate the brain metastasis of BC cells by recruiting myeloid cells [114, 115]. Additional information regarding the mechanisms of CCL2 production associated with BC brain metastasis is found in the section below.

## Mechanisms of CCL2 production in BC microenvironments

As described above, previous IHC studies indicated that both cancer cells and stromal cells, such as macrophages, fibroblasts, and ECs, are well-known sources of CCL2 in human BC tissues. There are at least three potential mechanisms for these cell types to be a source of CCL2: (1) BC cells constitutively produce CCL2, (2) BC cells produce CCL2 in response to stimuli present in the TME, and (3) stromal cells produce CCL2 in response to cancer cell products. Accumulating studies have indicated that all three mechanisms contribute to the production of CCL2 in BC microenvironments.

### Constitutive CCL2 production by BC cells

Nuclear factor of κB (NF-κB) is a transcription factor that regulates the transcription of many genes involved in inflammatory and immune responses. The promoter region of the human and mouse *CCL2/Ccl2* gene contains two NF-κB binding sites in the distal region, and they are particularly important for the induction of this gene in response to proinflammatory stimuli, such as LPS and tumor necrosis factor (TNF)-α [9, 116]. As presented in Fig. 1, many human BC cell types constitutively express CCL2 at various levels. Increased NF-κB binding activity has been detected in several human BC cell types, including MCF-7, MDA-MB-231, SK-BR-3, and BT-474 [117], suggesting a role for NF-κB in the process. Although the exact mechanisms regulating this constitutive CCL2 expression by BC cells remain unknown, several potential mechanisms have been identified.

#### Regulation by epigenetic alterations

The chromatin structure is plastic and dynamically changes through epigenetic processes that regulate the expression of many genes. There are three types of epigenetic alterations: DNA methylation, histone modification, and nucleosome positioning. DNA methylation occurs in C-G-rich sequences, known as CpG islands, in the promoter region of genes and inhibits their expression. Histone modifications are closely related to chromatin dynamics and regulate the transition between transcriptionally active euchromatin and inactive heterochromatin. Nucleosome positioning influences the accessibility of transcription factors and regulators to target DNA sequences and is affected by DNA methylation and histone modification. Several reports have indicated that epigenetic regulation of chemokine genes is associated with refractory diseases characterized by nonresolving inflammatory responses [118]. The epigenetic silencing of the CCL2 gene by DNA methyltransferase 1 (DNMT1)-mediated DNA methylation in the enhancer region of the gene has been shown to enhance the development of small cell lung cancer by repressing the infiltration of cytotoxic macrophages [119].

The corepressor CoREST1 regulates the localization and activity of histone-modifying enzymes, including lysine-specific demethylase 1 and histone deacetylase 1. Although several CoREST1-associated proteins have been reported to enhance BC progression, the role of CoREST1 in BC remains unclear. Mazumdar et al. reported that knockdown of CoREST1 in MDA-MB-231 cells significantly reduced the incidence and diminished the size of tumors compared to controls in mouse xenograft studies. There was a marked decrease in angiogenesis in tumors of CoREST1-knockdown cells, and CoREST1 knockdown resulted in a decrease in the secretion and expression of factors related to angiogenesis and inflammation, including VEGF-A and CCL2. These results led the authors to conclude that the epigenetic regulator CoREST1 promotes tumorigenesis in a BC model at least in part by regulating the expression of genes with profound effects on ECs and inflammatory cells in the tumor microenvironment [120]. However, it is not clear whether CoREST1 knockdown directly causes an epigenetic alteration of the *CCL2* gene.

#### Regulation by microRNA

MicroRNAs (miRNAs) are a family of non-coding RNAs with an average length of 22 nucleotides that act as posttranscriptional repressors of gene expression [121]. Aberrant expression of miRNAs is a hallmark of several diseases, including cancer, and microRNA expression profiling is associated with tumor development, progression, and response to therapy, suggesting their possible use as diagnostic, prognostic, and predictive biomarkers. Furthermore, since miRNAs can function as potential oncogenes or tumor suppressor genes, miRNA-based anticancer therapies have been exploited, either alone or in combination with current targeted therapies [122]. miRNA-binding sites are usually located in the 3ʹ-untranslated region (UTR) of mRNAs [121]. *CCL2* mRNA possesses a short 3′-UTR with only 373 base pairs; thus, a low number of miRNA-binding sites are predicted for CCL2 [123]. Nevertheless, miRNAs, such as miR-124 [124], can target *CCL2* mRNA. Downregulation of *CCL2*-targeting miRNAs may result in increased CCL2 production by BC cells. Alternatively, miRNAs targeting other genes may indirectly increase CCL2 production by BC cells.

Enhancer of zeste homolog 2 (EZH2) is the catalytic core subunit of polycomb repressive complex 2 (PRC2) capable of silencing target genes through trimethylation of H3K27 in histone H3 [125]. A high level of EZH2 is implicated in tumorigenesis and correlates with poor prognosis in various cancers, including BC [126]. Wang et al. first found that EZH2 inhibitor-treated BC cells, including 4T1 and MDB-MB-231 cells, enhanced M2 macrophage polarization in vitro and the tumor infiltration of M2 macrophages and BC cell lung metastasis in vivo, whereas EZH2 knockdown showed the opposite effects. Mechanistically, inhibition of EZH2 histone methyltransferase activity alone by EZH2 inhibitors in BC cells could reduce the enrichment of H3K27me3 on the *CCL2* gene promoter and increase *CCL2* transcription and CCL2 protein secretion, contributing to the induction of M2 polarization and recruitment of TAMs in TMEs. In contrast, knockdown of EZH2 resulted in DNA demethylation and subsequent upregulation of miR-124-3p levels, which inhibited the expression of its target *CCL2* in tumor cells, causing arrest of M2 polarization of TAMs [127]. Thus, high expression of EZH2 in BC cells could lead to increased CCL2 production via activation of the *CCL2* gene promoter and downregulation of miR-124-3p.

Zhang et al. reported an indirect influence of miRNA on the expression of *CCL2* in BC cells. miR-126/miR-126* derived from a single precursor directly targeted *Cxcl12* mRNA and suppressed the expression of *Ccl2* in a CXCL12-dependent manner in 4T1 cells [128]. It remains unclear how CXCL12 regulates the expression of CCL2 in BC cells.

#### Long noncoding RNA (lncRNA)

lncRNAs are defined as transcripts of more than 200 nucleotides that are not translated into proteins. They comprise a heterogeneous class of intergenic transcripts, enhancer RNAs (eRNAs), and sense or antisense transcripts that overlap other genes. lncRNAs have been reported to carry out diverse functions, including transcriptional regulation in *cis* or *trans*, organization of nuclear domains, and regulation of proteins or RNA molecules. Additionally, some transcripts that are annotated as lncRNAs have been demonstrated to actually encode small proteins [129].

Wang et al. [115] identified that the expression of a lncRNA, named lncRNA associated with BC brain metastasis (BCBM) (Lnc-BM), is a prognostic factor of the progression of brain metastasis in BC patients. In preclinical murine BC models, elevated Lnc-BM expression drove BCBM, while depletion of Lnc-BM with nanoparticle-encapsulated siRNAs effectively treated BCBM. Lnc-BM increased JAK2 kinase activity to mediate oncostatin M- and IL- 6-triggered STAT3 phosphorylation. In BC cells, Lnc-BM promoted STAT3-dependent expression of ICAM1 and CCL2, which mediated vascular cooption and recruitment of macrophages in the brain, respectively. Recruited macrophages in turn produced oncostatin M and IL-6, thereby further activating the Lnc-BM/JAK2/STAT3 pathway and enhancing BCBM. These results indicated that Lnc-BM and JAK2 promoted BCBMs by mediating communication between BC cells and the brain microenvironment.

#### Regulation by Twist1

Increased expression of the transcription factor Twist1 is associated with metastasis and poor survival in many human cancers, including BC. The prominent role of Twist1 in tumor progression is to induce EMT and extracellular matrix degradation. During EMT, Twist1 promotes stationary epithelial cells to lose cell‒cell junctions and gain migratory and invasive capacities. Interestingly, Twist1 is also implicated in vascularization both during development and in tumor models. During human embryoid body formation, Twist1 is co-upregulated with several genes regulating vascular development. Under hypoxia, Twist1 expression in tumor cells is directly induced by hypoxia-inducible factor (HIF)-1α and -2α. Finally, Twist1 expression promotes vascularization in tumor xenograft models. These observations suggest that Twist1 is involved in tumor angiogenesis [130].

Tumor angiogenesis may be modulated by tumor-infiltrating myeloid cells, such as macrophages and neutrophils. Low-Marchelli et al. aimed to uncover a novel function of Twist1 in recruiting macrophages to facilitate angiogenesis. To examine whether Twist1 can promote angiogenesis, the authors stably expressed Twist1 in immortalized human mammary epithelial cells (HMLEs) and examined their angiogenic potential using a quantitative chick chorioallantoic membrane (CAM) angiogenesis assay. The expression of Twist1 in HMLEs was sufficient to promote angiogenesis. A previous study demonstrated that Twist1 directly upregulated *CCL2* expression in white adipose tissue [131]. An experiment using a cytokine array detected increased levels of CCL2 in conditioned media from Twist1-transfected HMLEs (HMLE-Twist1), and *CCL2* mRNA in HMLE-Twist1 cells was 10.2-fold higher than in control cells. Direct induction of *CCL2* transcription was specific to Twist1 because another EMT-inducing transcription factor, Snail, induced CCL2 by only 1.6-fold. Expression of Twist1 in MDA-MB-468 human BC cells also markedly induced the expression and production of CCL2. Knockdown of Twist1 in several BC cell lines expressing high levels of Twist1, including mouse 168FARN and 4T1 and human SUM1315, reduced the level of *CCL2/Ccl2* expression. Thus, Twist1 expression was sufficient to induce CCL2 in normal and cancer mammary epithelial cells [130], and the expression of Twist1 may account for the elevated *CCL2* expression in BC cells with mesenchymal characteristics (Figs. 2 and 3).

#### Regulation by dysadherin

The prometastatic activity of dysadherin, a cancer-associated membrane glycoprotein, has been attributed to its ability to downregulate E-cadherin expression [132]. Nam et al. investigated the role of dysadherin in breast carcinogenesis. Dysadherin was highly expressed, particularly in more aggressive ER-negative BCs and cell types, including MDA-MB-231 cells. Knockdown of dysadherin, unexpectedly, reduced invasion through Matrigel in both E-cadherin-positive (MCF10Ca1a) and E-cadherin-negative cells (MDA-MB-231 and MDA-MB-435LV/Br). These results led them to hypothesize that dysadherin promotes cancer cell invasion by a novel mechanism that is independent of E-cadherin expression. A global gene expression analysis of dysadherin-knockdown MDA-MB-231 cells by siRNA identified *CCL2* as the transcript most affected by dysadherin knockdown. There was a more than 10-fold reduction in *CCL2* mRNA levels and a 7-fold decrease in CCL2 protein levels secreted into the cell culture supernatants of MDA-MB-231 cells transfected with dysadherin siRNA. Overexpression of dysadherin in T-47D cells, which express relatively low levels of endogenous dysadherin, resulted in a 2-fold increase in CCL2 secretion compared with that in mock-transfected cells. *CCL2* expression was upregulated by dysadherin partly through activation of the transcription factor NF-κB. The ability of dysadherin to promote tumor cell invasion in vitro was dependent on a CCL2 autocrine loop, and CCL2 secreted by dysadherin-positive tumor cells also promoted EC migration in a paracrine fashion. Finally, knockdown of CCL2 in MDA-MB-231 cells reduced their ability to metastasize after tail vein injection in vivo without affecting cell growth. These results indicated that dysadherin has prometastatic effects that are independent of E-cadherin expression and suggested that CCL2 plays an important role in mediating the prometastatic effect of dysadherin in ER-negative BC [45]. As shown in Fig. 1, T-47D cells express a lower level of CCL2 mRNA than MDA-MB-231 cells; thus, there is a correlation between dysadherin and CCL2 expression levels.

#### Regulation by KLF15

The *Kruppel-like factor 15 (KLF15)* gene, encoding a transcription factor belonging to the Kruppel-like factor (KLF) family, has recently been reported as a tumor suppressor gene in BC; thus, downregulation of KLF15 can promote the development of BC [133]. Kanyomse et al. [73] investigated the role of KLF15 in TNBC development and the mechanisms whereby KLF acts as a tumor suppressor. The expression of *KLF15* was significantly downregulated in BC cells, such as BT-549 (expressing a high-level *CCL2* mRNA; Fig. 1) and MDA-MB-231 cells, and BC tissues, and the methylation of the *KLF15* gene promoter partially contributed to its downregulation. Exogenous expression of KLF15 induced apoptosis and G2/M phase cell cycle arrest and suppressed cell proliferation, metastasis, and in vivo tumorigenesis of TNBC cells. Mechanistically, KLF15 targeted and downregulated the expression of *CCL2* and *CCL7* in KLF15-expressing BT-549 cells. Moreover, transcriptomic and metabolomic analyses revealed that KLF15 was involved in key antitumor regulatory and metabolic pathways in TNBC. These results suggested that KLF15 acts as a tumor suppressor by downregulating *CCL2* and *CCL7* expression and indicated that KLF15 may be a prognostic biomarker for TNBC.

#### Regulation by β-catenin

β-Catenin is a key signal transducer of the canonical Wnt signaling pathway and plays an important role in many physiological and pathological conditions. Dysfunction of the Wnt/β-catenin signaling pathway has been implicated in several types of human cancers, including BC [134]. In pathological processes, including cancer, β-catenin accumulates in the cytoplasm and then translocates to the nucleus, where it binds transcription factors of the T-cell factor/lymphoid enhancer factor (TCF/LEF) family, leading to the activation of specific genes. The loss of E-cadherin expression and the translocation of β-catenin to the nucleus are frequently associated with the metastatic conversion of epithelial cells [135]. Mestdagt et al. investigated the potential regulation of CCL2 expression by the β-catenin/TCF pathway. CCL2 was expressed and produced by invasive BC cells (MDA-MB-231, BT-549, and Hs578T) that did not express E-cadherin but not by noninvasive BC cells (MCF-7 and T47D) expressing high levels of E-cadherin. The *CCL2* gene promoter was activated in BT-549 cells transfected with β-catenin and TCF-4 cDNAs, and *CCL2* mRNA levels were similarly upregulated. The level of *CCL2* mRNA was downregulated after transfection with a siRNA against β-catenin in both BT-549 and Hs578T cells. These results, therefore, indicated that CCL2 is a target of the β-catenin/TCF/LEF pathway in BC cells, which could play a key role in BC progression [136].

Zhang et al. investigated the effect and mechanism underlying β-catenin-induced tumor progression. The authors first examined cytokines secreted from β-catenin-overexpressing BT-549 cells (BT549/LV- β-Catenin) using a cytokine array and found that secretion of several cytokines, including CCL2, was upregulated in conditioned media from β-catenin-overexpressing BT-549 cells. Overexpression of β-catenin in BT-549, HCC1937, MCF-7, or ZR75-1 cells upregulated *CCL2* mRNA expression and CCL2 protein production. β-catenin upregulated *CCL2* mRNA expression at the transcriptional level through direct binding to a putative TCF4 binding motif (CACATCTGT) in the *CCL2* gene promoter in BC cells, leading to the enhanced infiltration and polarization of macrophages in vivo. β-Catenin-mediated CCL2 secretion forms a paracrine feedback loop between BC cells and macrophages, which in turn promotes BC stem cell properties and BC growth and metastasis. Furthermore, combined inhibition of CCR2 and β-catenin markedly suppressed BC growth, suggesting a promising strategy of CCL2 intervention for BC therapy [137].

#### Regulation by PTEN

Phosphatase and tensin homolog (PTEN) is a major negative regulator of the signaling pathway defined by PI3K, AKT, and mTOR that plays a key role in controlling a wide range of essential cellular processes, including cell proliferation, growth, survival, and metabolism. Accordingly, loss of function of PTEN is one of the most common events observed in many types of cancer [138]. Zhang et al. [114] analyzed the public gene expression profiles of clinical metastases from distinct organs and organ-specific metastases from mice injected with various types of cancer cells, including MDA-MB-231 cells, and found that PTEN mRNA was markedly downregulated in brain metastases compared to primary tumors or other organ metastases. Analyses of PTEN expression by IHC confirmed a significantly higher rate of PTEN loss in brain metastases than in unmatched primary BC. PTEN loss was also detected at a significantly higher frequency in brain metastases than in matched primary BC of an independent patient cohort. The authors used MDA-MB-231 cells stably expressing a doxycycline-inducible PTEN-coding sequence and selected subclones that selectively metastasize to the brain. Induction of PTEN 7 days post-intracarotid injection markedly extended the overall survival of brain metastasis-bearing mice. These results indicated that PTEN loss primed brain metastasis outgrowth after tumor cell extravasation and that PTEN restoration suppressed outgrowth.

Cytokine array analyses revealed markedly reduced CCL2 secretion in PTEN-expressing MDA-MB-231 cells that metastasized to the brain compared to controls, whereas PTEN knockdown increased CCL2 expression. Moreover, the overall survival of brain metastasis-bearing mice with CCL2-knockdown MDA-MB-231 cells was significantly extended compared to controls. Mechanistically, PTEN induction decreased NF-κB p65 phosphorylation and reduced CCL2 secretion, whereas PTEN knockdown increased p65 nuclear translocation and CCL2 expression. Furthermore, *CCL2* mRNA and CCL2 protein expression in brain-seeking tumor cells were inhibited by the NF-κB inhibitor pyrrolidine dithiocarbamate (PDTC). Thus, loss of PTEN led to CCL2 upregulation via activation of NF-κB in BC cells.

Regarding the function of CCL2, coculturing with BV2 microglial cells enhanced proliferation and inhibited apoptosis of BC cells, such as HCC1954 and MDA-MB-231 cells. In vivo, brain metastases of CCL2-knockdown MDA-MB-231 cells injected via the intracarotid artery had decreased infiltration of CCR2-expressing IBA11-positive myeloid cells, corresponding to their reduced proliferation and increased apoptosis detected by Ki67 and TUNEL staining, respectively. Furthermore, IHC staining for PTEN and CCL2 in human primary breast tumors and matched brain metastases revealed significantly higher CCL2 expression in brain metastases than in primary tumors. Importantly, severe PTEN loss in brain metastases corresponded to higher CCL2 expression, which significantly correlated with IBA11 myeloid cell recruitment. These findings validated that PTEN downregulation in brain metastatic BC cells contributes to CCL2 upregulation and subsequent IBA11 myeloid cell recruitment in clinical brain metastases [114].

Gu et al. also reported PTEN-mediated CCL2 expression. The miRNA miR-200b-3p delivered by BC exosomes was taken up by alveolar epithelial type II cells in the lung and directly targeted PTEN. Inhibition of PTEN by this miRNA further promoted the activation of the AKT/NF-κBp65 pathway and subsequently increased the expression of CCL2. Thus, PTEN can regulate the expression of CCL2 in not only BC cells but also alveolar epithelial cells [104].

#### Regulation by NOTCH

Activation of NOTCH is a hallmark of the TNBC/basal-like BC subtype and contributes to the pathogenesis of BC by affecting several cellular processes, including CSC maintenance, cell fate specification, differentiation, proliferation, motility, and survival [139]. As described above, Tsuyada et al. reported that CCL2 induced NOTCH activation in BC cells [64]. In contrast, Shen et al. and Jaiswal et al. reported that activation of NOTCH upregulated CCL2 expression in basal-like BC and TNBC cells, respectively [140, 141]. In addition to BC cells, NOTCH has been demonstrated to regulate *Ccl2* transcription in mouse bone marrow cells as described below [142].

#### Regulation by HER2

Compared to basal-like or HER2^+^ subtype BCs, basal-like BCs with HER2 expression have been reported to be associated with worse prognosis [143, 144]. Furthermore, patients with both EGFR and HER2 expression show poorer prognoses than other groups of BC patients [145]. You et al. [146] first found that coexpression of EGFR and HER2 correlated with poor survival among BC patients and that overexpression of HER2 promoted the invasion and proliferation of EGFR^+^ MDA-MB-231 cells. This led to the hypothesis that proteins secreted by HER2-expressing BC cells may be responsible for increased proliferation and invasion. In fact, the level of CCL2 was significantly increased in HER2-overexpressing MDA-MB-231 cells at both the mRNA and protein levels. Increased CCL2 production was also detected using EGFR^+^ Hs 578T cells. Unexpectedly, however, treatment with trastuzumab, a HER2-neutralizing antibody, had no effect on the expression of *CCL2* mRNA, whereas neratinib, a pan-HER inhibitor, downregulated the expression of *CCL2* mRNA and secretion of CCL2 by HER2-overexpressing MDA-MB-231 cells. Knockdown of HER2 by HER2-specific siRNA downregulated the endogenous secretion of CCL2 by MDA-MB-453 (by approximately 35%) and BT-474 cells (by approximately 40%). Increased CCL2 production enhanced the migration of TAMs (M2-differentiated THP-1 cells), and TAMs treated with the culture supernatant of HER2-overexpressing MDA-MB-231 cells showed increased mRNA levels of protumor cytokines, including IL-8 and IL-1β. These results strongly suggested that the coexpression of EGFR and HER2 is important for upregulated CCL2 production by BC cells which recruits TAMs and induces CXCL8 and IL-1β production.

### CCL2 production by activated BC cells

#### Activation by TNFα

TNFα is a potent proinflammatory cytokine and promotes the development and progression of various cancers [147]. TNFα is present in high concentrations in the breast tumor/stroma milieu [148] and upregulates CCL2 production in 4T1 cells in vitro [51]. Bauer et al. found that MDA-MB-231 cells produced CCL2 in response to TNFα stimulation, and apigenin, a known anti-inflammatory constituent of parsley, inhibited TNFα-mediated CCL2 production by blocking inhibitor of nuclear factor kappa B kinase subunit epsilon (IKBKE). The results suggested the presence of TNFα-mediated CCL2 production by BC cells in BC microenvironments, and this TNFα effect could be attenuated by anti-inflammatory agents, including apigenin [149]. The levels of other proinflammatory cytokines, such as IL-6 and IL-1, are also elevated in BC patients [150], suggesting that these cytokines play a role in upregulated CCL2 production by BC cells.

#### Activation by TGFβ

Signals induced by TGFβ control various cell fates during development and tissue homeostasis; thus, dysregulation of this signaling pathway can drive several diseases, including cancer [151]. It is generally accepted that elevated TGFβ is tumor suppressive during early tumor outgrowth, whereas at later stages, there is a switch toward malignant conversion and progression. Inactivation of tumor suppressor genes, sequential acquisition of oncogenic mutations, and epigenetic alterations within the cancer genome redirect the growth inhibitory function of TGFβ toward activities that increase cell motility, invasion, and metastasis. In fact, in the majority of breast tumors and their metastases, nuclear phosphorylated Smad2 is detected, indicating an active TGFβ signaling pathway [152, 153].

Mandal et al. investigated the expression of CCL2 and CCL5 in tumor samples from 147 BC patients and examined the correlation of *CCL2* and *CCL5* expression with *TGF-β* expression. There was an inverse correlation of *TGF-β* expression with *CCL2* and *CCL5* mRNA levels in the early stages of BC. In contrast, in late stages, *CCL2*, but not *CCL5*, mRNA levels were found to be directly associated with *TGF-β* expression. TGF-β upregulated *CCL2* mRNA expression in MDA-MB-231 cells, whereas it downregulated the expression of both *CCL2* and *CCL5* in MCF-7 cells. A significant change in the Th1-Th2 ratio toward Th2 was observed within the primary tumors expressing moderate to high levels of *CCL2* and low to negative levels of *CCL5*. The CCL2-CCR2 interaction induced monocytes/macrophages to secrete the Th2-attracting chemokine CCL22 in vitro, suggesting that CCL2 secreted in the tumor microenvironment may attract and interact with monocytes/macrophages and favor Th2 T-cell accumulation by inducing CCL22 secretion. In a 4T1 BC model, administration of a TGF-β inhibitor significantly decreased *CCL2*, *CCL5*, and *CCL22* mRNA levels and reduced lung metastases. These findings collectively indicated that TGF-β regulates *CCL2* and *CCL5* expression in a stage-dependent manner during BC progression, which in turn determines the Th1-Th2 balance within the tumor microenvironment [154].

Gorbacheva et al. studied the mechanisms of TGF-induced *CCL2* gene transcription in MDA-MB-231 and HCC1937 BC cells representing a mesenchymal-like phenotype induced by TGF-β. Using bioinformatics, deletion screening, and point mutagenesis, the authors identified transcription factor (TF) binding sites in the human *CCL2* gene promoter and candidate TFs. Among these factors, only the knockdown of early growth response protein 1 (EGR1) and retinoid X receptor alpha (RXRA) reduced *CCL2* promoter activity in response to TGF-β. These factors also bound to the *CCL2* promoter in a TGF-β-dependent manner in a chromatin immunoprecipitation assay, and point mutations in the EGR1 and RXRA binding sites completely abolished the effect of TGF-β. Thus, TGF-β regulates the transcription of the *CCL2* gene through binding of EGR1 and RXRA to the *CCL2* gene promoter [155].

#### Activation by estrogen

Estrogens play a significant role in the development and progression of BC, and approximately two-thirds of all BCs are ER-positive. Blocking the action of estrogens with inhibitors, such as tamoxifen, for 5 to 10 years is a cornerstone in current BC therapy [156]. However, current anti-estrogen therapy causes severe adverse effects; nearly 50% of these cancers are intrinsically resistant to the therapy, and the majority of recurrences have maintained ER expression. Identifying the estrogen-regulated signaling pathways that could potentially serve as new targets for the treatment and prevention of ER-positive BC is needed. Svensson et al. investigated whether estradiol affects secreted levels of CCL2 and CCL5 in breast tissues. The extracellular in vivo levels of CCL2 and CCL5 in 10 postmenopausal BC patients (in microdialysates) were three to five times higher in cancerous tissues than in adjacent normal tissues, and a significantly increased number of macrophages were found in cancerous tissue compared with normal tissue. The levels of CCL2 and CCL5 in breast tissues significantly decreased after 6 weeks of tamoxifen therapy, whereas the levels in fat tissues were not changed. This result was verified by an ex vivo study demonstrating that normal human breast tissue biopsies exposed to tamoxifen exhibited significantly decreased levels of CCL2 and CCL5. In experimental BC models (tumor cells from MMTV-PyMT mice and MCF-7 cells), estradiol enhanced macrophage influx and angiogenesis through increased release of CCL2, CCL5, and VEGF. These effects were inhibited by anti-human CCL2 or CCL5 (R&D Systems) treatment, which resulted in potent inhibition of cancer growth. In addition, estradiol-induced the protumor activation of macrophages. In a zebrafish model, macrophages increased cancer cell dissemination via CCL2 and CCL5 in the presence of estradiol, which was inhibited with anti-CCL2 and anti-CCL5 treatment, indicating the potential of novel therapies targeting CCL2 and CCL5 [157].

Han et al. evaluated the levels of CCL2 under estradiol exposure in different BC cells and studied the roles of the CCL2-CCR2 axis in estrogen-induced effects on BC cells both in vitro and in vivo. ER-positive MCF-7 and T47D cells showed higher protein and mRNA levels of CCL2 in the presence of 17β-estradiol (E2), and tamoxifen could partially attenuate the effects, while no significant CCL2 induction was found in ER-negative MDA-MB-231 or SK-BR-3 cells. *CCL2* mRNA expression positively correlated with Twist (a regulator of EMT) immunostaining and aggressiveness of BC cells. Estrogen exposure facilitated the proliferation, invasion, and metastasis of hormone-dependent BC cells and promoted angiogenesis via the increased secretion of CCL2 in vitro and in vivo, which could be inhibited by the CCR2 antagonist RS102895. Knockdown of Twist in MCF-7 cells significantly inhibited E2-induced CCL2 production, indicating an essential role of Twist in CCL2 regulation under estrogenic conditions, which appeared to be regulated by the PI3K/Akt/NF-κB pathway. These results suggested that the CCL2-CCR2 axis may serve as a novel and much-needed therapeutic target for hormone-dependent BC [158].

#### Activation by S100 calcium binding protein A14 (S100A14)

The S100 family proteins possess a wide range of intracellular and extracellular functions and have been implicated in tumorigenesis and tumor progression [159]. Li et al. reported that S100A14 promoted the migration and invasion of BC cells in vitro and lung metastasis in vivo using mouse 4T1 and human MDB-MB-231 cells. By analyzing the S100A14-regulated transcriptome and proteome in S100A14-overexpressing 4T1 and control cells, S100A14 was found to regulate the expression of a panel of inflammatory chemokines and cytokines, including CCL2. Mechanistic studies showed that S100A14 upregulated the transcription of the *CCL2* and *CXCL5* genes via activation of NF-κB. To further examine the relevance of the S100A14-CCL2/CXCL5 axis in BC, the expression of S100A14, CCL2, and CXCL5 was examined in 55 human BC samples. Tumors with high S100A14 expression levels exhibited strong staining for CCL2 and CXCL5 by IHC. Serum S100A14 levels were positively correlated with CCL2 and CCL5 levels. Analyses of the GEO database showed that the increased expression levels of *S100A14*, *CCL2*, or *CXCL5* in primary BC were a determinant of poor metastasis-free survival in BC patients, suggesting that targeting S100A14 may inhibit aberrant CCL2/CXCL5 signaling in metastatic BC [160].

### Mechanisms of CCL2 production by stromal cells

Since unstimulated stromal cells do not produce a significant amount of CCL2, it is likely that tumor stromal cells, including TAMs and fibroblasts, are activated by interacting with BC cells. Since the disruption of tumor-stromal cell interactions could potentially inhibit tumor progression by reducing the production of tumor-promoting mediators, such as MMPs, cytokines, and chemokines, identifying the mechanisms whereby cancer cells manipulate host stromal cells for their advantage is important. The following studies provide insights into the mechanisms that upregulate CCL2 production by stromal cells in BC microenvironments.

#### Activation of macrophages by MDA-MB-231 cells

Fujimoto et al. examined in vitro whether BC cells have the potential to activate macrophages for CCL2 production by using human MDA-MB-231 cells and mouse thioglycolate (TG)-induced peritoneal macrophages. When macrophages or MDA-MB-231 cells were cultured alone, *CCL2/Ccl2* mRNA expression was low. However, when mouse macrophages were cocultured with MDA-MB-231 cells, *Ccl2* mRNA expression in macrophages was upregulated, suggesting that the interaction between BC cells and macrophages augments CCL2 production by TAMs [34].

#### Activation of macrophages by 4T1 cell products

As described above, in the 4T1 murine BC model, nontumor stromal cells, including macrophages, were the major sources of CCL2 [161]. To identify the potential mechanisms by which CCL2 production is upregulated in macrophages infiltrating 4T1 tumors, we analyzed the crosstalk between murine 4T1 cells and TG-induced mouse inflammatory macrophages and identified 4T1 cell-derived GM-CSF/CSF2 as a potent inducer of CCL2 production by inflammatory macrophages. Interestingly, unlike LPS, which upregulates a wide variety of proinflammatory genes in macrophages via Toll-like receptor (TLR) 4, GM-CSF upregulated the expression of a unique set of genes, including CCL2 and CCL7, suggesting that cancer cell-derived GM-CSF plays a critical role in the progression of 4T1 BC by inducing the production of CCL2 by TAMs. BC-derived GM-CSF is reported to regulate arginase 1 in myeloid cells to promote an immunosuppressive tumor microenvironment [162]. Contrary to our hypothesis, neutralization of GM-CSF in 4T1 tumor-bearing mice or transplantation of *Gmcsf*^-/-^ 4T1 cells did not alter the serum CCL2 level or tumor *Ccl2* mRNA expression level. Furthermore, the expression of *Ccl2* mRNA was still detectable in TAMs by ISH. Thus, tumor cell-derived GM-CSF was dispensable for the overall production of CCL2 in the 4T1 model [9, 161, 163]. We later noted that TAMs surrounding necrotic foci in 4T1 tumors were associated with high levels of *Ccl2* mRNA by ISH, and supernatants of necrotic 4T1 cells upregulated CCL2 production by TG-induced macrophages in vitro, suggesting that molecules released from necrotic cancer cells are also involved in the production of CCL2 and likely other cancer-promoting factors present in TMEs [164].

#### Activation of fibroblasts by 4T1 cell-derived platelet-derived growth factors (PDGFs)

Fibroblasts are another component of the tumor stroma, and those activated by growth factors and associated with cancer are termed CAFs. CAFs express several markers, including α-smooth muscle actin (αSMA), fibroblast activation protein, and PDGF receptors. Activation by growth factors, such as TGFβ, PDGFs, and fibroblast growth factor-2, results in the production of cytokines and chemokines important for cancer progression [165].

Potter et al. cocultured T47D or MDA-MB-231 cells with stromal cells (fibroblasts) derived from normal breast tissues or BC tissues. Higher CCL2 production was found when BC cells were cocultured with stromal cells from BC, suggesting the presence of crosstalk between BC cells and fibroblasts [166].

CCL2 is the product of the gene *JE*, one of the immediate early response genes induced in fibroblasts by PDGFs [167], and several human BC cell types have been shown to produce PDGFs [168], leading us to test the hypothesis that activation of fibroblasts by 4T1 cell-derived PDGFs contributes to overall CCL2 production by 4T1 tumors. When NIH-3T3 cells or mouse primary fibroblasts were cocultured with 4T1 cells or stimulated with the culture supernatants of 4T1 cells (4T1-sup), CCL2 production by fibroblasts markedly increased. As expected, 4T1 cells expressed mRNA for three PDGF subtypes, PDGF-a, b and c. To evaluate the role of PDGFs in 4T1-sup-induced CCL2 production by fibroblasts, we used a PDGF receptor antagonist, crenolanib. Pretreatment of fibroblasts with crenolanib almost completely inhibited 4T1-sup-induced CCL2 production by fibroblasts in vitro. However, treatment of mice with the PDGF receptor antagonists crenolanib or trapidil failed to reduce CCL2 production in 4T1 tumor-bearing mice. Histologically, 4T1 tumors contained a small number of αSMA^+^ CAFs, and *Ccl2* mRNA was not associated with CAFs by ISH. From these results, we concluded that although 4T1 cells have the capacity to activate fibroblasts via PDGFs, this crosstalk is just one of many mechanisms that elevates CCL2 production and subsequent cancer progression in this model [164]. Thus far, we have used only 4T1 cells to study the crosstalk between BC cells and stromal cells. Since BC is a highly heterogeneous disease and BC cells in each breast tumor may use different means to communicate with stromal cells for their advantage, it will be important to use additional BC cell lines to further identify molecules used for the crosstalk between BC cells and stromal cells.

#### Activation of mesenchymal stem cells (MSCs) by TNFα or IL-1β secreted from stromal cells and BC cells

As already described, TNFα and IL-1β are involved in various pathological processes, such as chronic inflammation, autoimmunity, and cancer [169, 170], and both cytokines are highly relevant to the inflammatory condition of breast tumors. They are minimally expressed by normal breast epithelial cells, but their expression by epithelial cells is detected in approximately 85% of BCs, and elevated expression of TNFα and IL-1 β is highly correlated with the relapse and progression of the disease [35]. The level of TNFα is significantly elevated in the serum of BC patients, and TNF-α is used clinically as a marker for tumor extension and outcome of BC [171, 172]. TNFα is secreted by stromal cells, mainly by TAMs, and cancer cells [148]. Katanov et al. reported that TNF-α and IL-1β induced the release of CCL2 by MSCs and CAFs generated in vitro by prolonged stimulation of MSCs with tumor-conditioned media of MDA-MB-231 and MCF-7 cells. BC patient-derived CAFs also released CCL2 in response to TNFα and IL-1β. CCL2 release by TNFα-stimulated MSCs was mediated by TNF-RI and TNF-RII and the NF-κB pathway. Supernatants of TNFα-stimulated MSCs showed chemotactic activity against THP-1 monocytic cells, which was dependent on CCL2. These results emphasized the important roles of inflammatory interactions in the breast tumor stroma. Although BC cells have been suggested to be the source of TNFα, its cellular sources have not been identified [36]. Liubomirski et al. also detected increased CCL2 production by cocultures of MDA-MB-231 cells and MSCs/CAFs stimulated with TNFα or IL-1β in vitro, partly dependent on direct physical contact between cancer cells and CAFs [173].

#### Activation of fibroblasts by BC cell-derived CXCL1/2/8

Forkhead box C1 (FOXC1) is a transcription factor with essential roles in mesenchymal lineage specification and organ development during normal embryogenesis. Consistent with these developmental properties, mutations that impair the activity of FOXC1 result in heritable Axenfeld-Rieger syndrome and other congenital disorders [174]. Overexpression of FOXC1 has been found in several types of cancer, and elevated expression of FOXC1 is proposed to be a critical marker for TNBC/basal-like BC and a prognostic indicator for TNBC lung metastasis [175]. Han et al. [176] compared gene expression profiles in control (low FOXC1 expression) and FOXC1-overexpressing MDA-MB-231 cells using microarray assays. A group of chemokines, including *CXCL1/2/8/10* and *CCL20*, were found to be among the top upregulated genes in FOXC1-overexpressing cells. *CXCL1/2/8* expression induced by FOXC1 in TNBC cells was partially mediated by NF-κB and potentially by direct activation of their promoters by FOXC1. The authors speculated that CXCL1/2/8 proteins stimulate lung fibroblasts to secrete certain factors and found that CXCL1/2/8 induced the expression of *CCL2* and *CCL7* in fibroblasts from the lung but not from other sources. Lung fibroblast-derived CCL2 and CCL7 potentiated the synthesis of cholesterol, which is necessary for angiogenesis and subsequent lung metastasis formation. Interestingly, *CXCL1/2/8* were among the top upregulated genes induced by CCL2 or CCL7 in FOXC1-overexpressing MDA-MB-231 cells. Thus, there appears to be a positive feedback regulatory loop of chemokines mediating the interaction between lung fibroblasts and neighboring tumor cells in TNBC lung metastases.

The association between FOXC1 and chemokine expression was also demonstrated using hepatocellular carcinoma (HCC) cells [177]. Unlike in BC cells, in HCC cell lines, CXCL8 activated the expression of FOXC1 via the PI3K/AKT/hypoxia-inducible factor 1α pathway. FOXC1 expression led to the transactivation of the *CXCR1* and *CCL2* genes, promoting inflammation and the invasive and metastatic abilities of HCC cells. It would be interesting to assess whether a similar mechanism also plays a role in BC cells.

#### Activation of adipocytes by BC cells

During obesity, adipocytes can secrete significant amounts of cytokines, including TNFα, IL-6, CXCL8 and CCL2. Furthermore, obesity is identified as a negative prognostic factor for BC [178]. Fujisaki et al. isolated adipocytes from BC (cancer-associated adipocytes; CAAs) and normal breast (normal breast adipocytes; NBAs) tissues and cocultured them with MCF-7 or MDA-MB-231 cells. A higher level of CCL2 was detected in the culture supernatants of CAAs cocultured with BC cells than NBAs cocultured with BC cells, and adipocyte-derived CCL2 promoted the migration of the two BC cell lines in a transwell cell migration assay [179]. The mechanism of BC cell-NBA interaction was unidentified.

#### Regulation by FBXW7 mutation in bone marrow cells

F-box and WD repeat-domain containing 7 (FBXW7, also known as FBW7, SEL-10, HCdc4, or HAgo) is the F-box protein component of a Skp1–Cul1–F-box protein–type (SCF-type) ubiquitin ligase, in which it acts as a receptor for substrate recognition. Most FBXW7 substrates are promoters of cell growth, including c-MYC, NOTCH, cyclin E, c-JUN, KLF5, and mTOR; therefore, FBXW7 may serve as a tumor suppressor. Analysis of FBXW7 expression revealed that approximately 6% of primary human tumors harbored mutations in this gene. Findings obtained by genetic analyses of mice in which the *Fbxw7* gene was conditionally deleted in various tissues supported an important role for FBXW7 in the suppression of tumorigenesis [142].

Previous studies of FBXW7 focused on its functions in tumor cells, and little is known about the role of this protein in stromal cells in the host microenvironment. Yumimoto et al. orthotopically transplanted several mouse cancer cell types, including EO771 BC cells, into mice in which the *Fbxw7* gene was conditionally deleted in bone marrow cells (*Mx1-Cre Fbxw7*^*Δ/Δ*^ mice). Lung metastasis was found to be enhanced in mice lacking FBXW7 in bone marrow compared with control mice. Mechanistically, the absence of FBXW7 resulted in NOTCH accumulation and consequent activation of *Ccl2* gene transcription in bone marrow-derived suppressor cells (BMSCs). The increased production of CCL2 by these cells likely promoted the formation of metastatic niches through the recruitment of MDSCs of monocyte lineages (Mo-MDSCs) and macrophages. Inhibition of CCL2/CCR2 signaling with a CCR2 inhibitor, propagermanium [180], reduced the number of metastases in FBXW7-deficient mice. These results suggested that the FBXW7/NOTCH/CCL2 pathway in BMSCs plays a central role in the regulation of cancer metastasis [142]. The finding that FBXW7 is an upstream regulator of CCL2 expression also has implications for the development of new treatment strategies for cancer patients [181].

#### Regulation by β3-integrin in mural cells

Increased formation of blood vessels (BVs) is thought to drive tumor growth through elevated nutrient delivery; however, it remains unclear whether mural cells, including vascular smooth muscle cells and pericytes, can directly affect tumor growth independent of BV functions. Wong et al. provided clinical data correlating high percentages of mural-β3-integrin-negative tumor BVs with increased tumor sizes without changes in BV numbers. Mural-β3-integrin loss in implanted and spontaneous mouse tumor models enhanced tumor growth with no detectable effects on BV numbers or function. At a molecular level, the loss of β3-integrin in mural cells enhanced the FAK-HGFR-Akt-p65 pathway and elevated the production of CXCL1, CCL2, and tissue inhibitor matrix metalloproteinase-1. In particular, mural cell-derived CCL2 stimulated tumor cell MEK1-ERK1/2-ROCK2-dependent signaling and enhanced tumor cell survival and tumor growth. These data indicated that mural cells can control tumor growth via paracrine signals, such as CCL2, regulated by β3-integrin, providing a previously unrecognized mechanism of cancer growth control [182].

## Biodistribution of CCL2 by cancer cell-derived exosomes

Cancer cell-secreted vesicles, especially exosomes, are implicated in guiding metastatic dissemination, with specific surface composition that determines some aspects of organ-specific distribution. However, the mechanisms whereby exosomes are distributed to distinct organs remain unknown. Lima et al. provided a novel mechanism whereby BC cell-derived exosomes can be distributed to remote organs, such as the lung, to promote metastasis in human BC patients and mouse BC models [183]. BC cell-derived exosomes were bound by cytokines present in the TME, such as CCL2. CCL2 bound to exosomal GAG side chains of proteoglycans, such as CD44, HSPG2, syndecan-1, and versican. After conjugation, BC cell-derived exosomes were predominantly retained in organs, such as the lung, and preferentially taken up by CCR2^+^ immune cells, such as MDSCs and NK cells. These immune cells, which interact with cytokine-conjugated exosomes, contribute to the formation of a metastasis-favorable environment and promote subsequent metastatic progression.

## Negative regulation of CCL2 activity by CCL2-binding receptors

### CCRL2 expressed on BC cells

CC-chemokine receptor-like 2 (CCRL2) is a 7-transmembrane receptor that resembles chemokine receptors. Although the binding of some chemokines, including CCL2, to this receptor has been reported, its role as a chemokine receptor has not been established [184]. Wang et al. found that three minimally invasive cell lines, MCF-7, T-47D, and BT-474 cells, expressed high levels of CCRL2, while two highly invasive cell lines, MDA-MB-231 and BT-549 cells, expressed low levels. Moderately invasive MDA-MB-468 cells expressed a moderate level of CCRL2. Stable overexpression of CCRL2 in MDA-MB-231 and BT-549 cells attenuated the chemotaxis and invasion stimulated by its proposed ligand CCL2. CCRL2 inhibited CCL2-induced p38 MAPK phosphorylation and upregulated the expression of E-cadherin. This effect was eliminated by an inhibitor of p38 MAPK. Stable overexpression of CCRL2 also inhibited the growth of MDA-MB-231 cells both in vitro and in vivo, suggesting that CCRL2 functions as a tumor suppressor in human BC cells and that its expression may have prognostic significance in human BC [185].

### ACKR2 expressed on BC cells

Altered lipid metabolism is known to be involved in BC pathogenesis, but the underlying mechanism in lipid metabolism-mediated metastasis of BC remains unknown. Adipocytes have the capacity to produce CCL2 and promote cancer metastasis, and patient survival has been shown to be associated with the expression of atypical chemokine receptors/chemokine decoy receptors [186]. Zhong et al. were interested in the mechanism that may influence cancer metastasis and patient prognosis, particularly in patients with altered lipid metabolism. Based on a large amount of evidence from previous studies, they proposed a hypothesis that patients with increased expression levels of atypical chemokine receptor 2 (ACKR2) receptors to which CCL2 binds have less chance for tumor metastasis, whereas patients with decreased ACKR2 expression but high levels of CCR2^+^ monocytes are more likely to develop metastasis and have worse outcomes. However, in patients with lower ACKR2 expression levels but elevated levels of CCR2^+^ NK cells, primary tumors can be suppressed and present better outcomes [187]. Further studies are needed to test this hypothesis.

## CCL2 as a biomarker for BC

### Single-nucleotide polymorphisms (SNPs)

As already described in this review, the promoter region of the human *CCL2* gene contains multiple *cis*-elements for various transcription factors. Two NF-κB sites in the distal region of this gene are particularly important for induction of this gene in response to proinflammatory stimuli, such as LPS and TNF-α [9]. Rovin et al. identified two single-nucleotide polymorphisms (SNPs) located at positions –2518 (G or A) and –2076 (A or T) from the major transcriptional start site of the *CCL2* gene. A biallelic G/A polymorphism at position -2518 of the 5′-flanking region influenced the transcriptional activity of the putative distal regulatory sequence of the gene. Furthermore, monocytes from individuals carrying a G allele at -2518 produced higher levels of CCL2 after IL-1β treatment than monocytes from individuals with the A/A allele [188]. Since its discovery, the -2518A/G polymorphism in the *CCL2* gene has been extensively investigated to evaluate its association with the development of diseases, such as Alzheimer’s disease, but there are no consistent results [189]. Ghilardi et al. reported that the presence of at least one G allele in patients with stage I or II BC at the time of diagnosis enhances their risk of metastasis by a factor of 2.67 compared with patients diagnosed in the same stage but homozygous for allele A. However, they could not confirm any associations between the presence of a *CCL2* G allele and vascular or lymph node invasion. Although their findings supported their hypothesis, the authors concluded that the results must be considered carefully and that additional studies are needed to confirm the role of the functional *CCL2* gene SNPs in BC. Furthermore, functional *CCL2* gene SNPs represent only one of many factors involved in determining the prognosis of BC. If their data are confirmed, the *CCL2* gene SNPs could be reliable candidates for inclusion in a panel of genetic risk factors influencing the course of the disease [190].

### Plasma CCL2 and IHC CCL2 expression levels

The plasma CCL2 level in healthy individuals is low; however, it rises quickly in response to i.v. injection of LPS [191]. It has been reported that increased serum or plasma CCL2 levels are associated with many inflammatory diseases [192]. Lebrecht et al. aimed to evaluate whether CCL2 serum levels could serve as a tumor marker for breast diseases. The authors measured CCL2 serum levels in 135 patients with BC, 30 with DCIS I–III, and 143 with benign breast lesions and in 27 healthy women and examined whether the value of CCL2 serum levels can serve as a differentiation marker between malignant, preinvasive and benign breast diseases and as a predictive marker for the biological phenotype of BC. Median CCL2 serum levels in the three groups of patients and healthy women were 200 (57–692) pg/ml, 194 (58–525) pg/ml, 174 (39–529) pg/ml, and 175 (67–425) pg/ml, respectively; thus, there were no differences among the groups. In BC patients, increased CCL2 serum levels were correlated with advanced tumor stage (*p* = 0.04) and lymph node involvement (*p* = 0.04), but serum CCL2 levels could not be established as a differentiation marker between malignant and benign breast tumors [193].

Wang et al. compared the concentrations of 10 chemokines and chemokine receptors (CXCL5, 7, 8, 12, CCL2, CXCR4, CCR2, 5, 7 and atypical chemokine receptor DARC/ACKR1) in the serum of 148 patients diagnosed with benign breast changes, epithelial proliferation (present only or with atypia), in situ carcinoma and invasive carcinoma. In all cases from benign disease to invasive carcinoma, the concentrations of CXCL8, CXCR4 and CXCL12 were significantly different. In the benign subgroups (benign change, benign change with proliferation, atypia), the concentrations of CCL2 and CCR5 were significantly different. In invasive carcinoma cases, the DARC concentration was significantly correlated with the relapse risk of patients. From these results, the authors concluded that changes in chemokine and receptor concentrations in sera contribute to the evolution of primary BC [194].

The authors expanded their study by investigating the expression of 13 chemokines and chemokine receptors by IHC in five BC subtypes, namely, HER2, basal-like, luminal A, luminal B, and normal breast-like. According to IHC of 205 BC tissues, the level of CCL2 staining was significantly different between the different BC subtypes and was negatively associated with ER and PR expression. Kaplan‒Meier analysis showed that a low level of CCL2 staining was associated with better outcomes in BC patients [37].

Heiskala et al. studied the frequency of TAMs and CCL2-producing cells in three groups of primary tumor-recurrence pairs, where recurrence was recorded within 2 years (Group 1), between 5 and 10 years (Group 2), and after 10 years (Group 3), based on IHC. All established BCs were heavily infiltrated by CD68^+^ cells. Both in primary and in recurrent lesions, the infiltration was more abundant in the peritumoral than in the intratumoral stroma. The mean frequency of TAMs positive for CD14 or CD163 in the intratumoral stroma and CCL2^+^ tumor epithelial cells was higher in recurrences than in the corresponding primary tumors. In primary tumors, a high frequency of CD14^+^ cells and a high level of CCL2 by tumor epithelial cells were associated with early recurrence. From these results, the authors proposed that high frequencies of CCL2^+^ tumor epithelial cells and CD14^+^ TAMs are significant risk factors for rapid tumor recurrence [195].

Lubowicka et al. investigated the applicability of the plasma levels of CCL2, CCR2 and the commonly accepted tumor marker CA 15-3 (also known as MUC1) in the diagnosis of BC. They measured the plasma concentrations of three proteins in 3 groups of patients: (1) BC patient group; (2) benign breast tumor group; and (3) control group of healthy women. The median levels of CCL2 in the entire BC group were significantly higher than those in the control groups, similar to the median levels of CA 15-3. CCR2 was a negative marker, and its levels were significantly lower in the BC group than in the healthy group. The concentration of CCL2 in BC patients increased with advancing tumor stage, while the median level of CCR2 decreased with advancing stage. CCL2 showed the highest value of sensitivity (64.95%) in the entire BC group and in early stages of the disease. The highest specificity was obtained by CA 15-3. In the early stages of BC, the highest area under the ROC curve (AUC) of all tested parameters was observed for CCL2 or CCR2 (stage I: 0.6604 and 0.6564, respectively; stage II: 0.7768 for CCR2). The findings of this study suggested that CCL2 and CCR2 may be applicable in the diagnosis of BC patients, particularly in combination with CA 15-3 [196].

### Tumor *CCL2* mRNA levels

**Fig. 5:**
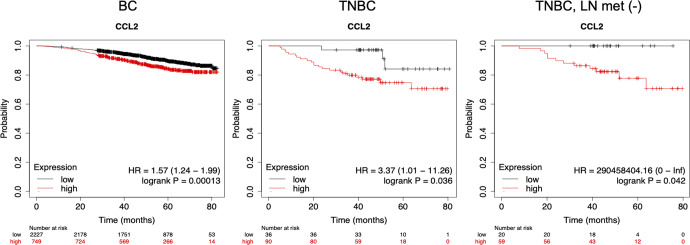
Association between *CCL2* mRNA expression and overall survival (OS) of patients with BC, TNBC and TNBC without lymph node metastasis by the Kaplan‒Meier Plotter [197]. Plots were generated with the RNA-seq data at https://kmplot.com/analysis/. An auto-cutoff was used

The correlation between the tumor *CCL2* mRNA expression level and survival was analyzed in some of the studies introduced in this review. In general, high *CCL2* mRNA expression was correlated with poor prognosis. We analyzed the association between the level of tumor *CCL2* mRNA expression and patient prognosis using the existing microarray or RNA-seq database (Kaplan‒Meier Plotter [197]). When microarray data were analyzed, there was no significant correlation between *CCL2* expression level and overall survival (OS). However, when mRNA sequence data were analyzed, higher *CCL2* mRNA expression was associated with a poor OS in all patients with BC (HR 1.57) and patients with TNBC (HR 3.37), particularly with TNBC without lymph node metastasis (HR 290458404.16) (Fig. 5). This suggests that BC patients with high *CCL2* mRNA expression are more likely to develop metastases in distant organs, such as the lung.

## Conclusions

**Fig. 6:**
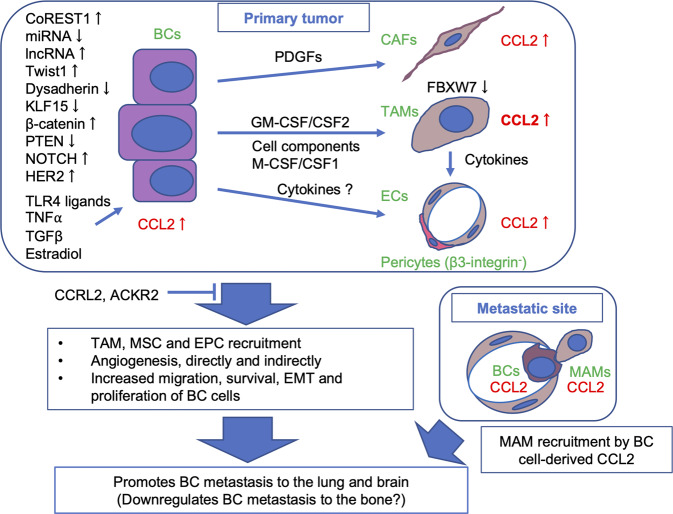
CCL2 produced in primary BC tumors promotes BC progression. CCL2 production by BC cells is increased  by several mechanisms that involve CoREST1, miRNA, lncRNA, Twist1, dysadherin, KLF15, β-catenin, PTEN, NOTCH and HER2, and in response to stimuli, such as TLR4 ligands, TNFα, TGFβ, and estrogen. CCL2 is also produced by stromal cells, including macrophages, fibroblasts, ECs and pericytes, after activation by products of cancer cells and stromal cells. Decreased expression of FBXW7 in Mo-MDSCs and macrophages results in increased CCL2 production. Macrophages are the major CCL2-producing cells in the TME. Produced CCL2 promotes the recruitment of additional macrophages, mesenchymal stem cells (MSCs), and endothelial precursor cells (EPCs), and angiogenesis. Additionally, CCL2 promotes BC cell migration, survival, EMT, and proliferation, leading to BC progression, especially lung and brain metastasis. CCL2 produced at the metastatic site by both cancer cells and macrophages plays an important role in the metastatic seeding of BC cells. Downregulation of lung and bone metastasis by BC cell-derived CCL2 has also been reported, but further clarification is needed

Since its discovery in 1989, many studies have been conducted to define the biological role of tumor-derived CCL2 and the mechanisms regulating CCL2 production during the development and progression of BC both in vitro and in vivo (Fig. 6). Although some disagreements exist among the studies, one consistent finding is that CCL2 is a critical chemokine that promotes BC progression by recruiting macrophages, such as TMAs and MAMs, into primary tumors and lung metastases. TAMs are one of the major sources of CCL2, and CCL2 produced and released by stromal cells is functionally important. TAM-derived CCL2 and VEGF stimulate angiogenesis, allowing cancer cells to enter the circulation. Cancerous epithelial cells also express and produce various levels of CCL2, and cancer cell-derived CCL2 facilitates the recruitment of MAMs that assist the extravasation and growth of BC cells at the metastatic site. There are only a few studies evaluating the role of cancer cell-derived CCL2 in metastasis to the bone and brain. Several studies have demonstrated evidence that MCP-1 directly acts on cancer cells via CCR2 and enhances survival, proliferation, and migration/invasion, contributing to the progression of BC; however, these CCL2 effects could not be recapitulated in studies using genetically engineered mouse models. The importance of cancer cell-derived CCL2 may be further clarified by using CCL2-deficient BC cells. Several mechanisms have been reported to account for the increased constitutive expression of CCL2 by BC cells, but the relative importance of each mechanism remains unclear. Thus, additional studies are required to better understand the mechanisms by which CCL2 promotes BC development and progression.

Although the contribution of CCL2 to BC metastasis, especially lung metastasis, is rather clear, antagonists of CCL2 or its receptor CCR2 have not been successfully used in the clinical setting [24, 198, 199]. To prevent metastasis, long-term treatment with antagonists may be needed, which makes the use of CCL2/CCR2 antagonists more difficult. Aggravation of lung metastasis after the termination of anti-CCL2 Ab treatment observed in a mouse study raised an alarm. Future studies should be directed to determine at which stage of BC development administering such antagonists is most effective. It will also be important to consider using them with other treatments, including surgery, radiation and anticancer drugs, although necrotic cancer cells may induce inflammatory responses and upregulate CCL2 production. Regarding the use of CCL2 as a biomarker for BC, there is no clear conclusion at present. Evaluating the level of *CCL2* mRNA in tumors may be more useful than evaluating serum CCL2 levels. We hope that the knowledge obtained from all studies in the past and future will lead us to a better understanding of the mechanisms of BC progression and new effective treatments for patients suffering from BC.
